# Sumac Polyphenols as Pan-Herpesvirus Inhibitors

**DOI:** 10.3390/ijms262110398

**Published:** 2025-10-26

**Authors:** Shavkat I. Salikhov, Yuliya I. Oshchepkova, Jamolitdin F. Ziyavitdinov, Jamshid M. Ashurov, Nodir S. Berdiev, Mikhail S. Kolundin, Akhmed O. Gaidarov, Ali S. Turgiev, Kirill I. Yurlov, Victor F. Larichev, Irina T. Fedyakina, Valeria L. Andronova, Natalia E. Fedorova, Alla A. Kushch, Alexander V. Ivanov, Eduard V. Karamov

**Affiliations:** 1 Sadykov Institute of Bioorganic Chemistry of the Academy of Sciences of Uzbekistan, Tashkent 100125, Uzbekistan; info@biochem.uz (S.I.S.);; 2 Gamaleya National Research Center for Epidemiology and Microbiology of the Russian Ministry of Health, 123098 Moscow, Russia; 3 Engelhardt Institute of Molecular Biology, Russian Academy of Sciences, 119991 Moscow, Russia

**Keywords:** herpes simplex virus type 1, herpes simplex virus type 2, SARS-CoV-2, influenza A virus, Epstein–Barr virus, cytomegalovirus, polyphenols, sumac, geranium

## Abstract

Pandemic preparedness is a complex of threat-agnostic countermeasures developed in advance which would be efficient against a future outbreak regardless of its causative agent, and broad-spectrum antivirals constitute a critical component of this complex. Plant polyphenols are known to suppress viruses of unrelated families by acting on multiple viral and cellular structures. We therefore searched for broad-spectrum antivirals among polyphenols that have been confirmed as safe to humans. The ellagitannin geraniin and galloylglucose constituents of the drug Rutan (1,2,3,4,6-penta-O-galloyl-β-D-glucose [R5], 3-bis-O-galloyl-1,2,4,6-tetra-O-galloyl-β-D-glucose [R6], 2,4-bis-O-galloyl-1,3,6-tri-O-galloyl-β-D-glucose [R7], 2,3,4-bis-O-galloyl-1,6-di-O-galloyl-β-D-glucose [R8]) were isolated from *Geranium sanguineum* and sumac (*Rhus coriaria*), respectively. We revealed their activity towards herpes simplex viruses (HSV-1 and HSV-2), human cytomegalovirus (CMV), and the Epstein–Barr virus (EBV). R5 suppressed HSV-1 and HSV-2 with equal efficiency, while Rutan and R7 were more active against HSV-1, and geraniin against HSV-2. Rutan and R5 also inhibited the intracellular replication of CMV and EBV (contrary to our expectations, geraniin and polyphenols R6–R8 showed no activity). Thus, we have shown for the first time that sumac polyphenols are capable of suppressing—in addition to HIV, influenza virus, and SARS-CoV-2—the reproduction of representatives of all three Orthoherpesviridae subfamilies, meeting the criteria for further development as broad-spectrum antivirals.

## 1. Introduction

Viral infections currently pose the greatest pandemic threat due to the high transmissibility and replication rate of pathogens. Despite increasing investments in epidemic prevention and prior experience of countering recent outbreaks of the SARS-CoV, MERS-CoV, Ebola, and Zika viruses, the world turned out to be unprepared for the COVID-19 pandemic that caused >6 mln deaths and unprecedented economic loss [1]. One of the major lessons of this pandemic is that the spread of an infection cannot be stopped without efficient antiviral drugs. Vaccines cannot fully substitute antivirals, as vaccination is ineffective in already infected patients, so may not provide defense against newly emerging viruses and their variants, and are often ineffective in patients with immunosuppression (e.g., due to high dose chemotherapy or after organ transplantations). Antivirals could alleviate the course of viral infection or even prevent it when taken prophylactically (pre- or post-exposure) by those who cannot be vaccinated [2]. They can also slow down the infection spread by decreasing the viral load in asymptomatic infected individuals and lead to a faster recovery in patients with pronounced symptoms of the disease, thus decreasing the number of patients requiring hospitalization and alleviating the burden on the healthcare system.

The etiological agent of coronavirus disease 2019 (COVID-19), severe acute respiratory syndrome coronavirus (SARS-CoV-2), is just one of many pandemic threats. There are more than 250 zoonoses with pandemic potential [3], such as dengue virus, which has infected an estimated 400 million people in more than 128 countries, and the Venezuelan equine encephalomyelitis virus. However, approved antivirals are available to treat infections caused by viruses from ten families only. Thus, the development of efficient chemotherapeutic agents against existing and emerging viral infections is highly warranted [4].

The predicted presence in natural reservoirs of about one million unknown viruses that can once establish an infection in humans [5] makes it impossible to rapidly develop effective agents for those numerous pathogens. An alternative is the search for broad-spectrum antivirals that would be active against viruses of different families by targeting a process common to their life cycles (which is contrary to direct-acting antivirals). This approach is referred to as threat-agnostic and is used by the Biomedical Advanced Research and Development Authority (BARDA) of the Department of Health and Human Services in the US and by the European Health Emergency Preparedness and Response Authority of the EU.

The majority of viruses on the list of dangerous pathogens of National Institute of Allergy and Infectious Diseases (NIAID)—*Variola vera*, arenaviruses, bunyaviruses, flaviviruses, filoviruses, henipaviruses, coronaviruses, etc.—are enveloped, and their entry into the target cells occurs via membrane fusion (either on the cell surface or within endosomal compartments). Despite the diversity of viral fusogenic proteins, the basic biophysical and biochemical features of the fusion process are common to all enveloped viruses. Therefore, one approach to developing broad-spectrum antivirals may be to search for potent membrane fusion inhibitors [6]. It has been successfully validated by the development of peptide fusion inhibitors (active against HIV, MERS-CoV, HCoV-OC43, and SARS-CoV-2) [7,8], lipopeptides (active against coronaviruses and HIV) [9], defensin peptide (active against influenza A virus [IAV] and several strains of SARS-CoV-2) [10], ginkgolic acid (active against IAV, Zika, Ebola, and measles viruses) [11], estrogen receptor ligands (panfilovirus inhibitors active against Ebola and Marburg viruses) [12], protoporphyrin IX derivatives (active against Lassa and Machupo viruses, IAV, and SARS-CoV-2) [13], perylenes [14], and diphylline [15]. Since viruses are obligate parasites [16], suppression of their reproduction can be achieved by affecting basic processes involving host cell machinery such as viral genome replication and virion formation [17]. In particular, DDX3 RNA helicase inhibitors are active against HIV, hepatitis C virus (HCV), dengue, and West Nile viruses [18,19]; microfilament GTPase blockers exhibit activity against respiratory viruses [20]; and a TIE-2 tyrosine kinase inhibitor (a diphenylurea derivative) was found to suppress SARS-CoV-2, adenovirus, human cytomegalovirus (CMV), herpes simplex virus (HSV), dengue, and influenza viruses [21].

Another group of antiviral agents is presented by plant polyphenols [22,23,24,25]. They include epigallocatechin gallate [26,27], pentagalloylglucose [28], ladanein (BJ486K) [29], silymarin [30], and many other compounds. These non-toxic compounds often inhibit replication of viruses of unrelated families [31,32]. Moreover, they can act on multiple targets in the life cycle of a virus [33,34], many of which are the proteins/processes of a host cell [35,36], which reduces the likelihood of resistance development [37,38,39,40,41]. As an example, many plant polyphenols target the cell entry of enveloped viruses, thus being able to act towards several infections with no risk of resistance development.

We have previously demonstrated that plant polyphenols are potent inhibitors of HIV [42], influenza virus [43], and SARS-CoV-2 [44,45]. Polyphenols of sumac (*Rhus coriaria*) are the active ingredients of Rutan, a drug developed by us and currently approved in the Republic of Uzbekistan for the treatment for influenza and COVID-19 [46]. To determine whether plant polyphenols are suitable for developing a threat-agnostic broad-spectrum antiviral, we have been examining their ability to inhibit the replication of DNA and RNA viruses that cause socially significant diseases.

In this paper we report antiviral activity towards viruses of Orthoherpesviridae family: HSV-1 and HSV-2, CMV, and the Epstein–Barr virus (EBV), representing Alphaherpesvirinae, Betaherpesvirinae, and Gammaherpesvirinae subfamilies, respectively. Orthoherpesviridae comprise over two hundred species, nine of which are pathogenic to humans [47]. Due to high prevalence (up to 100% of the human population is infected, depending on the country) and the virtually asymptomatic course of the diseases in individuals with non-compromised immunity, pathogenicity of members of Orthoherpesviridae has been neglected. Nevertheless, the global economic damage from genital herpes alone amounts to several dozen billion dollars [48]; highly resistant HSV strains for which the standard treatment regimen is ineffective are becoming increasingly widespread, and the eye and CNS lesions they cause lead to patient disability and death [49,50,51,52]. CMV infection may trigger neurodegenerative diseases [53,54] and malignant tumors [55,56]; ganciclovir, foscarnet, and cidofovir, approved for its treatment, are highly toxic, and their long-term use leads to the emergence of resistant virus strains [57,58]. Remote consequences of EBV infection also include neurodegenerative [59,60], oncological [61,62], and severe autoimmune pathologies [63,64]. In the absence of specific chemotherapy agents [65] and vaccines (which cannot be developed due to lack of an animal model) [66], patients with EBV infection are treated with toxic drugs (used for the treatment of HSV and CMV infections) that suppress EBV genome replication, but quickly induce resistance mutations, making long-term treatment ineffective. The development of an efficient prophylactic anti-EBV vaccine is estimated to prevent 200,000 cases of cancer annually. Thus, the search for and development of new drugs against Orthoherpesviridae is highly warranted.

In the current manuscript we demonstrate that sumac polyphenols not only exhibit activity against HIV, influenza virus, and SARS-CoV-2, but also inhibit human viruses of all three Orthoherpesviridae subfamilies.

## 2. Results

### 2.1. Selection of Polyphenols

The main criterion for including a plant polyphenol or polyphenol-rich extract in this study was the availability of previously obtained data (generated by us or others) on their antiviral activity (Table 1 and references therein). We also took into account information on the use of materials matching the main criterion as approved pharmaceuticals. As can be seen from the table, two pharmaceuticals were included in this study: Rutan, which is included in therapeutic regimens of influenza and COVID-19 patients in Uzbekistan, and geraniin, an anti-diarrhea remedy in Japan. In addition, since Rutan is a mixture of water-soluble sumac polyphenols, we decided to explore the antiviral activity of its four major components that account for 88.7% of the drug’s pharmacopeial substance (Table S1). Studies on the spectrum of antiviral activity were previously carried out only for one of the Rutan polyphenols, 1,2,3,4,6-penta-O-galloyl-β-D-glucose (R5 in Table 1); the activity of the other three polyphenols (R6, R7, and R8) was previously explored only against SARS-CoV-2 during Rutan repurposing for the treatment of COVID-19 [2].

### 2.2. Preparation of Polyphenols

#### 2.2.1. Rutan and Its Components

**Figure 1:**
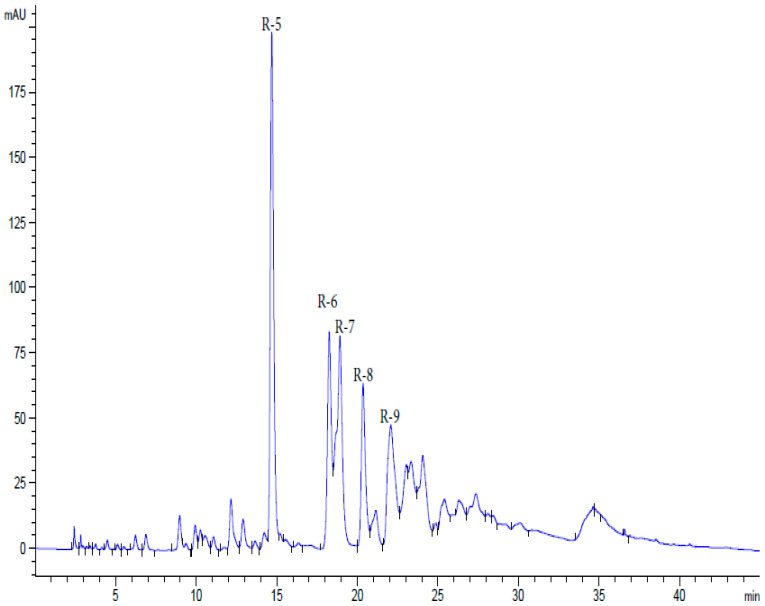
HPLC analysis of water-soluble polyphenols of sumac.

The pharmacopeial substance of the drug Rutan was obtained using a modification of the technology developed previously [44] (according to the procedure described in Experimental Section 4.2.1). Successive extraction of air-dried leaves of sumac (*R. coriaria*) grown in Uzbekistan, with 40% aqueous ethanol, chloroform, and ethyl acetate, followed by precipitation with chloroform, resulting in a complex of plant polyphenols with a yield of 15% of the original raw material weight. The sum of water-soluble polyphenols was isolated by fractioning using hydrophobic chromatography with a yield of 83% of the mass of the polyphenol complex (Figure 1). Water-soluble polyphenols were represented by five major components, R5, R6, R7, R8, and R9 in the ratio 39.2: 14.8: 26.3: 8.4: 6.7, respectively (Table S1).

**Figure 2:**
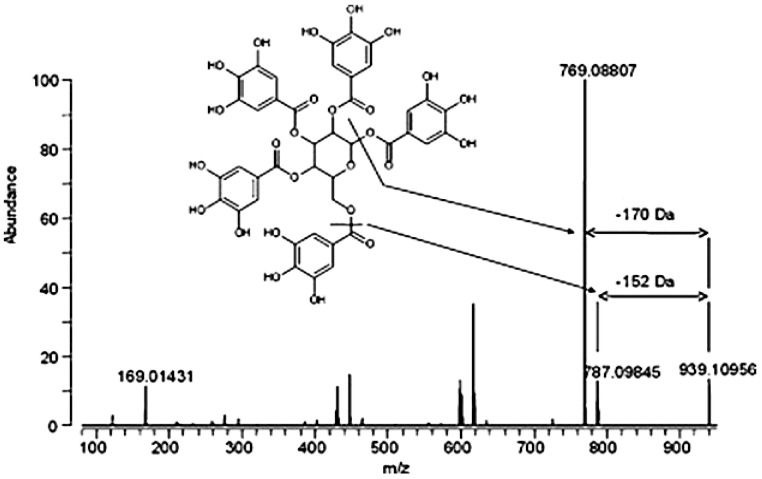
Polyphenol R5 of the pharmacopeial substance of Rutan: structure, MS/MS spectrum, and fragmentation pathways.

Figure 2 shows the MS/MS spectrum, structure, and possible fragmentation pathways of the molecular ion of polyphenol R5. Fragmentation of R5 (*m*/*z* 939.05) occurs via the cleavage of a fragment with *m*/*z* 169.8 (12%), which corresponds to the residue of gallic acid. The five peaks (varying in intensity) found in the spectrum after gallic acid cleavage correspond to fragments with *m*/*z* 769, 601, 447, 276.9, and 152. The presence of five additional peaks (787, 617, 465, 313, and 169) indicates that a fragment with *m*/*z* 152 is also cleaved. In all likelihood, fragmentation of the R5 molecular ion occurs at both the ester bond and the bond involving the carbonyl group of gallic acid (decreasing the fragments by *m*/*z* 170 and *m*/*z* 152, respectively).

The structures of polyphenols R5–R8, isolated by semi-preparative high-performance liquid chromatography (HPLC), were confirmed by mass spectrometry (Table 2, Figures S1–S8) and NMR spectrometry (see Section 4.2.1).

#### 2.2.2. Geraniin

**Figure 3:**
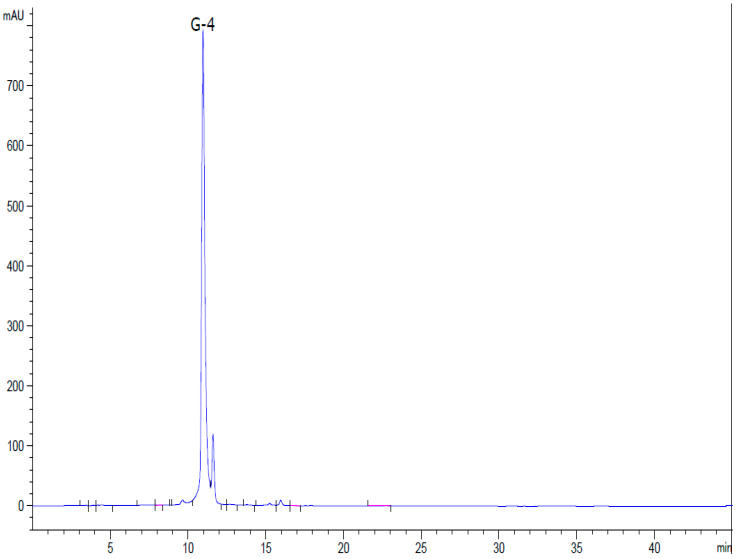
HPLC of geraniin.

Geraniin was obtained using the technology developed previously [120] (Section 4.2.2). Sequential extraction of the dried aerial parts of *Geranium sanguineum* (grown in Uzbekistan) with 40% aqueous ethanol and ethyl acetate, followed by precipitation with chloroform, allowed us to obtain a complex of the plant polyphenols with a yield of 4.67% of the original raw material weight. The fraction of water-soluble polyphenols was isolated by hydrophobic chromatography with a yield of 49% of the polyphenol complex weight. Geraniin was isolated from this fraction by semi-preparative HPLC with a yield of 75.5% of the total weight of water-soluble polyphenols and a purity of more than 95% (Figure 3).

In the mass spectrum, geraniin is represented by a deprotonated molecular ion with *m*/*z* = 951.4312 (Figure S9). Targeted MS/MS analysis allowed us to detect peaks of molecular ions [M-H]^−^ (*m*/*z* 951.4) and M-H_2_O (*m*/*z* 933.2), as well as daughter fragment ions (*m*/*z* 765.9, 614.2, 461.1, 301.05, 169.03, and 142.06) formed as a result of elimination of gallic acid residues and one ellagic acid residue (Figure S10).

**Figure 4:**
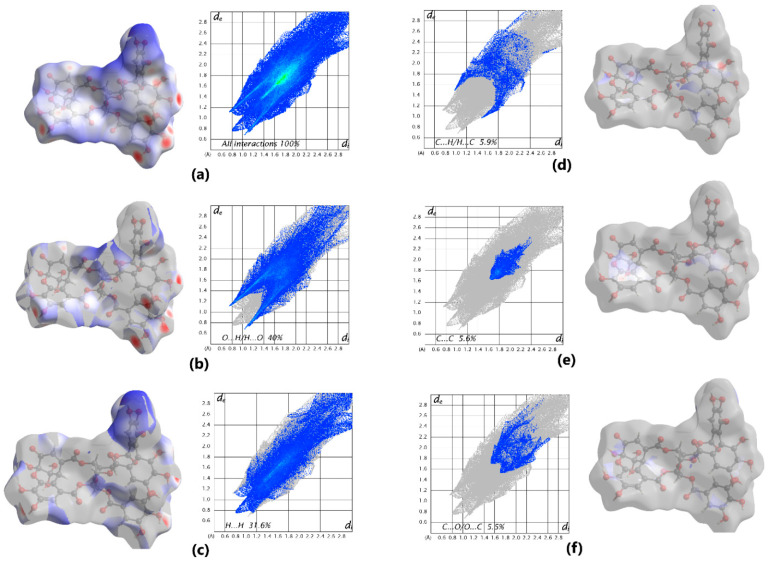
Hirschfeld surface mapped over d_norm_ and decomposed fingerprint plots for dominant interactions. In the fingerprint plot, the color scale denotes the relative frequency of interactions, from dark blue (low) to green/yellow (high). The densest (brightest) central zone at *d*_i_ ≈ 1.5–1.8 Å and *d*_e_ ≈ 1.5–1.9 Å indicates where intermolecular contacts occur most frequently.

The final structure of the component representing dehydroellagitannin geraniin was confirmed by X-ray diffraction. Previously, Luger et al. [121] reported that the crystal of geraniin hydrate form belonged to the orthorhombic syngony with the space group P2_1_P2_1_P2_1_, if analysis was performed at 120 K. Here we found that geraniin forms a monoclinic crystal system with the space group P2_1_. This new crystal hydrate form of geraniin was characterized by X-ray diffraction analysis at 290 K and Hirschfeld surface analysis (2D fingerprint plots were generated using the software package Crystal Explorer 21.5 [122]). It allowed us to visualize intermolecular interactions in the crystal lattice of geraniin (Figure 4a). Hirschfeld surface analysis showed that the classical O–H…O hydrogen bonds correspond to O…H/H…O contacts contributing 40% (Figure 4b) and represented by a pair of spikes. The contacts H…H contribute 31.6% (Figure 4c) and appear as prominent features on the Hirschfeld surface. Minor contributions come from the C…H/H…C (5.9%), C…C (5.6%), and C…O/O…C (5.5%) bonds, as shown in Figure 4d–f.

**Figure 5:**
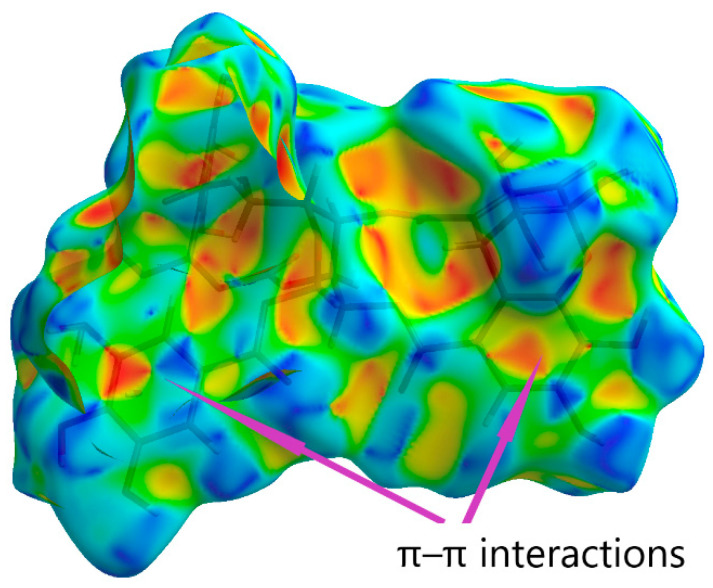
Hirschfeld surface constructed for geraniin using the shape index.

The Hirschfeld surface shape index serves as a tool for visualizing π–π stacking interactions. Adjacent red and blue triangles indicate the sites of π–π interactions (Figure 5).

**Figure 6:**
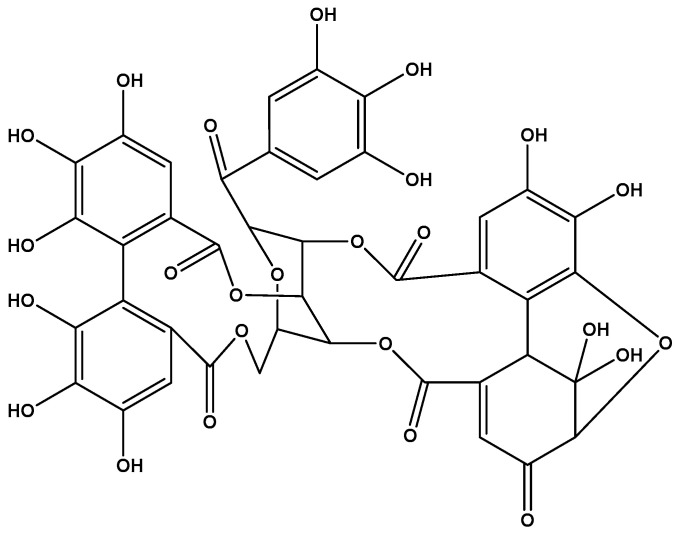
Structure of geraniin.

Thus, the isolated polyphenol was identified as the ellagitannin (1R,7R,26R,28S,29R,38R)-1,13,14,15,18,19,20,34,35,39,39-Undecahydroxy-2,5,10,23,31-pentaoxo-6,9,24,27,30,40-hexaoxaoctacyclo [34.3.1.0~4,38~.0~7,26~.0~8,29~.0~11,16~.0~17, 22~.0~32,37~]tetraconta-3,11,1 3,15,17,19,21,32,34,36-decaen-28-yl 3,4,5-trihydroxybenzoate (Figure 6).

### 2.3. Antiviral Activity of Polyphenols Against Respiratory Viruses and HIV-1

First, we performed biological testing of Rutan and geraniin against IAV and SARS-CoV-2. As earlier we already showed that Rutan exhibits anti-IAV activity, here we evaluated its components in IAV-infected MDCK cells. In addition, we evaluated geraniin against SARS-CoV-2 (in Vero E6 cells), and HIV-1 (in MT-4 cells) to confirm its activity. In all experiments three parameters were determined: concentrations of a drug that suppressed virus replication (IC_50_) or decreased cell viability (CC_50_) by 50% as well as their ratio—the selectivity index (SI, CC_50_/IC_50_ [123]). The results are summarized in Table 3, Table 4 and Table 5. Indeed, Rutan demonstrated activity against IAV, which was similar to the previously reported data [46]. Moreover, here we showed for the first time that its component R6 is almost inactive, R5 is moderately active, and components R7 and R8 exhibit the highest activity, with SIs of 191 and 233, respectively (Table 3). Judging by the SI values, R8 was 15 times more active against IAV than Rutan, but was still surpassed by the drug Oseltamivir (positive control).

The IC_50_ value for the preparation of geraniin under study (10.83 μg/mL; Table 3) is comparable with the results obtained earlier by Joo et al. (5.3 μg/mL) [72] and Choi et al. (34 μg/mL) [96] (neither group provided IC_50_ values for geraniin). Similarly, we found no difference in the anti-SARS-CoV-2 activity between our geraniin preparation (IC_50_ = 5.2 μg/mL; Table 4) and geraniin isolated from *Elaeocarpus sylvestris*, which was reported to suppress the virus replication in the concentration range of 1–25 μg/mL [80] (this preprint is the only publication describing the effect of geraniin on SARS-CoV-2 in cultured cells, but the authors do not provide the IC_50_ and SI values). Concerning HIV-1, our data for geraniin (IC_50_ = 1.6 μg/mL, SI = 25.6; Table 5) virtually coincide with the results published by Notka et al. (IC _50_ = 0.46 μg/mL, SI = 29.27) [103], even though the values of both parameters are much lower than those obtained for the antiretroviral drug AZT (used as a positive control).

Thus, biological testing showed that the antiviral activity of the preparations of Rutan and geraniin corresponds to data from the literature, which allows for their further evaluation against herpes viruses.

### 2.4. Activity of Polyphenols Against Viruses of the Family Orthoherpesviridae

#### 2.4.1. Herpes Simplex Virus

Next, we evaluated the activity of polyphenols against HSV-1 and HSV-2 in Vero E6 cells. Virus replication was assessed by quantifying virus-induced cytopathic effects (CPEs). For each compound, we measured concentrations at which they suppressed virus replication by 50% and 95% (IC_50_, IC_95_) or caused death in 50% cells (CC_50_). The results, summarized in Table 6, demonstrate that all polyphenol preparations, with the exception of R6 and R8, suppress dose-dependently the reproduction of HSV-1 and HSV-2. The activity of R6 (against both viruses) and R8 (against HSV-2) could be detected only in subtoxic concentrations. R5 suppressed both viruses with equal efficiency, while Rutan and R7 were more active against HSV-1, and geraniin against HSV-2. Judging by the SI values, geraniin was a considerably more powerful inhibitor of both viruses than foscarnet (a second-line drug for the treatment of herpes virus infections caused by resistant strains), which was the reference compound used as a positive control.

Thus, geraniin turned out to be the most active in inhibiting both viruses. Although the ability of this polyphenol to suppress HSV-1 and HSV-2 reproduction is well documented, and the results of the present work (Table 6) are generally consistent with reported data, a direct comparison makes no sense because of the large scatter in the published values of CC_50_ and IC_50_ [112,113,114,115,116,117]. The same conclusion is true for polyphenol R5, the activity of which against HSV-1 and HSV-2 has been repeatedly described [84,92,93]. The discrepancy between the published quantitative results is probably underlain by differences in the experimental setup and sources of polyphenols (and, consequently, in the composition/purity of the materials studied).

#### 2.4.2. CMV

Antiviral activity against CMV (strain AD169) was studied in two types of settings, which corresponded to prophylactic and therapeutic administration, differing in the sequence of addition of polyphenols and the virus to the human embryo lung fibroblast (HELF) culture. In the prophylactic scheme (pre-inoculation setup), the cells were first treated by the drug and then infected; in the therapeutic scheme (post-inoculation setup), a drug was added to the pre-infected cells. Pre-inoculation and post-inoculation setups were to reveal the effects of a polyphenol on (a) virus adsorption onto and entry into the cells and (b) post-entry steps of replication [124,125]. However, the pre-inoculation setup does not completely rule out the internalization of polyphenols and their subsequent effects on virus replication. Likewise, the post-inoculation setup cannot exclude the possibility that the effect of a drug is due to blocking the spread of the virus (though mechanisms of primary infection and cell-to-cell transmission of the infection are not identical [126,127], including in CMV [128]).

**Figure 7:**
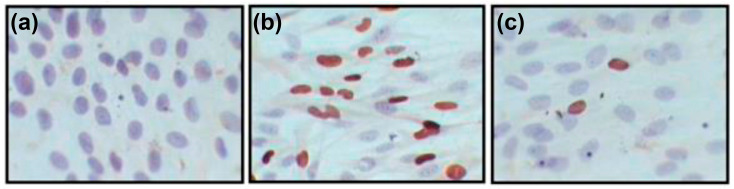
Immunocytochemical detection of CMV-infected HELF, using a mixture of monoclonal antibodies to the viral proteins IE1-p72 and pp65; Olympus light microscope (Japan), 800× magnification. (**a**) Uninfected cells; (**b**) infected cells; (**c**) cells infected with CMV following exposure to Rutan. Brown coloration in B and C corresponds to localization of viral proteins in CMV-infected cells. Cell nuclei (purple) are stained with hematoxylin.

To assess the effect of polyphenols on intracellular replication of the virus (post-inoculation setup), we determined the percentage of cells expressing viral proteins (stained with virus-specific monoclonal antibodies) (Figure 7).

**Figure 8:**
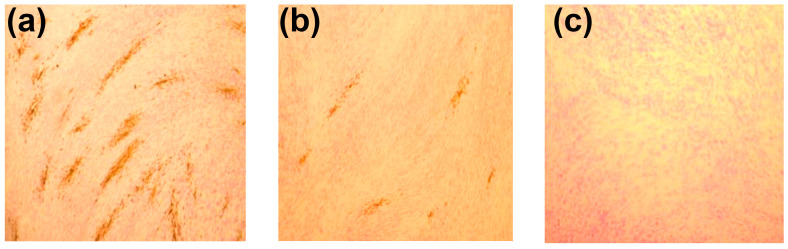
Detection of virus-specific PFU in HELF monolayers inoculated with CM from CMV-infected cells (**a**) or CMV-infected cells exposed to R5 (**b**). No PFU was found in HELF culture inoculated with CM from uninfected cells not exposed to R5 (**c**). Brown staining corresponds to the localization of clusters of infected cells containing CMV proteins visualized with monoclonal antibodies; 100× magnification.

In addition, we assessed the effect of polyphenols on the production of infectious virions. This was carried out by (i) collecting conditioned medium (CM) from CMV-infected HELF treated with polyphenols, (ii) using it for infection of new HELF, and (iii) counting the foci of infection 5 days later. The number of foci in cultures of CM-inoculated HELF reflects the amount of PFU (i.e., infectious virions [129]) in the CM (as exemplified by Figure 8).

**Figure 9:**
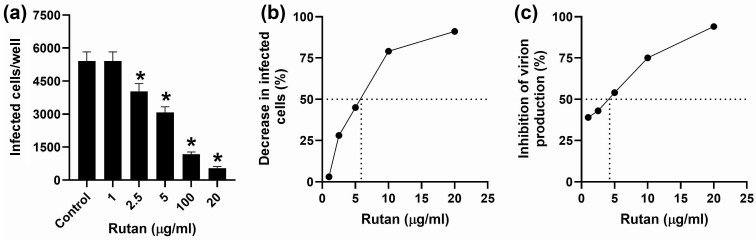
Rutan inhibits intracellular replication and production of infectious virions of CMV_AD169_. (**a**) Concentration-dependent reduction in the number of CMV-infected HELF cells. (**b**) Effect of Rutan on number of infected cells in HELF cultures (compared to untreated cells). (**c**) Effect of Rutan on number of infectious virions in the CM (compared to untreated cells). * *p* < 0.05 compared to the control.

Post-inoculation exposure to Rutan caused a tenfold decrease in the number of CMV-infected cells: the number of cells positively stained for viral proteins in HELF not exposed to the drug was 5593 ± 457 cells/well, while in HELF treated with 20 μg/mL Rutan their number was reduced to 542 ± 73 cells/well (Figure 9a). Statistically significant differences were also observed when Rutan was used at lower concentrations (Figure 9a,b). A 50% decrease in the number of infected cells was observed at 5.9 μg/mL Rutan (IC_50_), which corresponds to an SI value of 28.8 (with CC_50_ being 170 μg/mL; Table 7).

Exposure of CMV-inoculated HELF cultures to Rutan at 20, 10, and 5 μg/mL reduced the production of infectious virions by 94%, 75%, and 53.7%, respectively (Figure 9c). The calculated IC_50_ value was 4.0 μg/mL, which corresponds to an SI of 42.5 (taking into account the CC_50_ of 170 μg/mL)**.**

**Figure 10:**
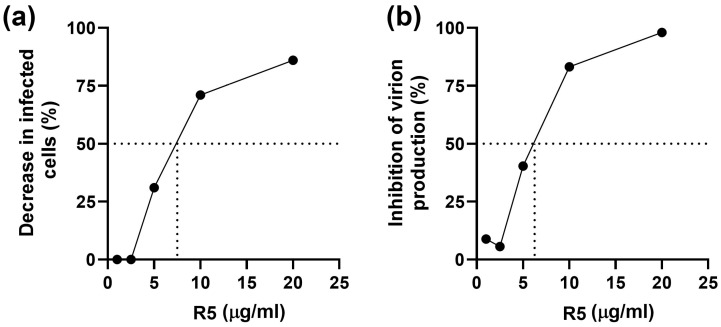
Suppression by polyphenol R5 of intracellular replication (**a**) and production of infectious virions (**b**) of CMV_AD169_.

Polyphenol R5, taken at concentrations of 10 and 20 μg/mL, effectively reduced the number of infected HELF by 71% and 86%, respectively (Figure 10a). The calculated IC_50_ value was 7.4 μg/mL, which corresponds to an SI of 20.5 (taking into account the CC_50_ of 150 μg/mL). Exposure of CMV-inoculated HELF cultures to R5 also decreased the production of infectious virions (Figure 10b). The calculated IC_50_ value was 6.4 μg/mL (SI = 23.4).

**Figure 11:**
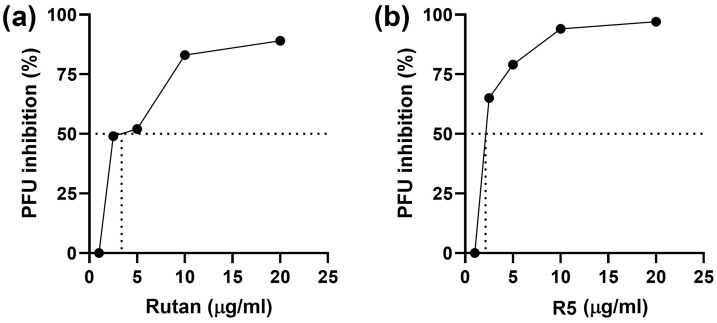
Suppression of CMV infection by Rutan (**a**) and polyphenol R5 (**b**) in the pre-inoculation setup. CMV_AD169_ strain (titer 300 PFU/mL) was used to infect HELF cells. The number of plaques in the cultures exposed to the polyphenols was counted and expressed as a percentage of the number of plaques in control cultures (not exposed to polyphenols).

In the pre-inoculation setup, both Rutan and R5 demonstrated pronounced antiviral activity (Figure 11): SI values were equal to 50 and 58, respectively, exceeding those obtained in the post-inoculation setup, and ganciclovir was inactive (Table 7). Thus, cell pretreatment with Rutan or R5 is beneficial, in spite of the fact that their activity in the post-inoculation setup was lower than that of ganciclovir.

Of note, our results for R5 differ from those reported by Kim et al. [35], who failed to detect the activity of 1,2,3,4,6-penta-O-galloyl-β-D-glucose against CMV. This may be due to the fact that the virus–cell system of those authors (CMV_Towne_ strain and human foreskin fibroblasts) differs from that used in the present work.

#### 2.4.3. EBV

**Figure 12:**
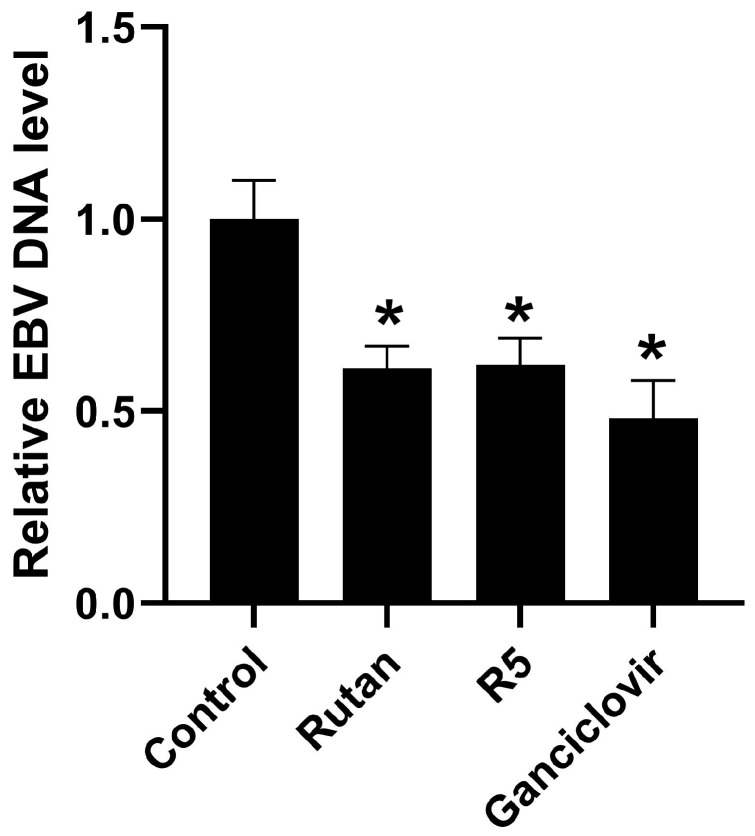
Effect of Rutan and polyphenol R5 on the amount of EBV DNA in B95-8 cells. * *p* < 0.05 compared to the control.

Effects of the polyphenols on EBV replication was assessed by the amount of viral genomic DNA in B95-8 cells, an immortalized B lymphoblastoid line derived from EBV-transformed B-lymphocytes of cotton-top tamarin (*Saguinus oedipus*) [130] (a monkey endemic to Ecuador [131]). Polyphenols were added to the cultured cells, and the cultures were incubated for 48 h. Ganciclovir (100 μg/mL) was used as a reference compound. The cells were then washed with pure medium, the DNA was isolated, and the amount of EBV genomic DNA was determined by real-time PCR. The data are shown in Figure 12. Exposure to Rutan (1 μg/mL) and R5 (10 μg/mL) for 48 h decreased the level of intracellular viral DNA by 38 ± 3% and 39 ± 5%, respectively. Thus, Rutan and R5 inhibit EBV replication in B95-8 cells to the same degree as ganciclovir, but in much lower concentrations (Figure 12). Other polyphenols showed no activity. The results on the cytotoxic and antiviral effects of the compounds are summarized in Table 8.

## 3. Discussion

Here we have demonstrated that Rutan exhibits promising activity towards various members of the Orthoherpesviridae family. Its efficacy in most cases exceeds the antiviral activity of its individual polyphenol components. It is worth noting the parameters of inhibition of CMV and EBV reproduction, i.e., viruses against which polyphenols R6–R8 do not show any activity, and R5 is clearly inferior to Rutan (Table 7 and Table 8). If R5 were the sole active ingredient of Rutan (polyphenols R6–R8 being inactive), the result would have been the opposite. So, it is highly likely that R6–R8 have no targets in the life cycles of CMV and EBV, but somehow contribute to the effect of R5 on its possible targets. The results of experiments with CMV suggest that there are at least two types of targets for R5 that confer both early and central/late stages of the virus life cycle. This is supported by activity of this substance in pre-inoculation and post-inoculation setups (Table 7).

It has already been mentioned above that the antiviral activity of R5 (1,2,3,4,6-penta-O-galloyl-β-D-glucose) has been extensively reported (Table 1). In particular, it was shown that one of the mechanisms of HSV-1 suppression by this polyphenol consists in disruption of the expression of viral tegument proteins and interference with nucleocapsid transport [93]. Considering the structural and functional similarity of tegument proteins in herpesviruses of the Alphaherpesvirinae, Betaherpesvirinae, and Gammaherpesvirinae subfamilies [132,133], we may speculate that inhibition of CMV and EBV reproduction by R5 involves the same or similar mechanism(s).

During entry steps, CMV binds to various types of cell surface receptors, one of which is the integrin α_V_β_3_ [134,135]. Exposure of cells to 1,2,3,4,6-penta-O-galloyl-β-D-glucose was reported to downregulate β_3_-integrins [136]. So, it is possible that the high antiviral activity of R5 in the pre-inoculation setup was due to a decrease in the expression of α_V_β_3_, caused by this polyphenol. However, a rather short pretreatment period may not be sufficient for the downregulation of expression. This merits further studies of molecular mechanisms by which Rutan and its components suppress replication of herpes viruses.

Various plant antioxidants were reported to exhibit antiviral activity against DNA and RNA viruses. One of the most widely known examples is epigallocatechin gallate (EGCG), which suppresses a wide array of viruses including herpesviruses [137,138], HBV [139,140,141], HCV [142], Zika [26] and chikungunya [143] viruses, human papilloma virus [144], and respiratory viruses [27,145,146,147]. In these cases, various mechanisms of action were proposed, such as the suppression of virus binding to receptors [27,142] and concomitant entry [26,145], targeting viral enzymes and regulatory proteins [35,138] and affecting levels of transcription of viral genes [141], regulation of signaling cascades [35,139] and gene methylation [147], affecting metabolic pathways such as lipid biosynthesis [146] or even targeting cell chaperones [148]. Other polyphenols were also reported to have multiple mechanisms of action against herpesviruses (e.g., [149]). Compounds that are structurally similar to Rutan components were also shown to affect a number of targets, previously not linked to the replication of viruses, with α-glucosidase being an example [150]. So, the identification of the molecular mechanism underlying Rutan’s antiviral activity against herpesviruses is a challenging task, especially taking into account the fact that Rutan suppresses both early and central/late stages of their life cycle.

Based on the discussion above, the contribution of polyphenols R6–R8 to the activity of Rutan against CMV and EBV may consist in the dysregulation of cell signaling pathways (transcription factors, kinases, etc.), which itself does not affect the replication of viruses, but increases the antiviral activity of R5.

The ability of Rutan and R5 to suppress the reproduction of herpesviruses belonging to all three subfamilies of the Orthoherpesviridae family suggests that those polyphenols are also active against other herpesviruses and may even be pan-herpesvirus inhibitors (to date, drugs with such a profile of pharmacological activity are unknown, but attempts to identify them are underway [151,152]).

Contrary to our expectations, geraniin—a polyphenol with a very broad spectrum of antiviral activity (Table 1)—did not interfere with CMV or EBV infection under the experimental conditions used. We did confirm its activity against both HSV-1 and HSV-2 (Table 6), which was high enough to warrant further studies in experimental animals and humans. Since ingestion of geraniin (a pharmaceutical in Japan) is proven to be safe [119], its repurposing for the treatment of herpes simplex infections may result in the development of formulations for systemic administration (not just ointments, sprays, and the like). This would be a major accomplishment, because there is an unmet need in drugs active against acyclovir-resistant HSV strains and/or capable of preventing infection [153]. Prior research demonstrated that geraniin may have several targets in the life cycle of HSV, including glycoproteins gB [113] and/or gD [113,116,117], as well as tegument proteins [116]. We plan to both address the mechanisms of anti-HSV activity in geraniin and assess its in vivo efficacy in HSV-infected animals.

## 4. Materials and Methods

### 4.1. Chemicals

Acetonitrile, methanol, trifluoroacetic acid (TFA), and formic acid were purchased from Sigma-Aldrich, St. Louis, MO, USA; chloroform and Na_2_SO_4_ from Khimprom, Novocheboksarsk Russia; DMSO from LabBase, Korea; acetone, acetic acid, and ethyl acetate from Navoiazot, Navoiy Uzbekistan; and ethanol from Biokimyo, Yangiyul Uzbekistan.

### 4.2. Isolation, Purification, and Identification of Polyphenols

#### 4.2.1. Rutan (Sum of Water-Soluble Polyphenols of Sumac Leaves, *Rhus coriaria* L.)

Extraction with 40% aqueous ethanol. Crushed air-dried sumac leaves (100 g) were placed in a 3 L flask equipped with a reflux condenser, 2.0 L 40% aqueous ethanol was added, and extraction was carried out in a water bath at 50–55 °C for 2 h with regular stirring. The extract was filtered through a Buchner funnel, and a new portion of the extractant was added to the raw material. After three iterations, the raw material was dried under a hood until traces of solvent were removed.

Extraction with chloroform. Raw material treated as described above with 40% aqueous ethanol (100 g) was placed in a 1.5 l flask equipped with a reflux condenser, 1.0 L chloroform was added, and extraction was performed as described above. The resulting extract was placed in a rotary evaporator and the chloroform distilled off. The aqueous residue was treated with ethyl acetate; the organic fraction was concentrated and treated with a 6-fold volume of chloroform. The resulting flocculent precipitate was the complex of sumac polyphenols.

Hydrophobic chromatography. The complex thus obtained (450 mg) was dissolved in 50 mL 2.5% aqueous ethanol and passed through a 1.5 × 30 cm column filled with Silochrom 80 C18 sorbent, using a peristaltic pump. Unsorbed substances were washed off with 2.5% aqueous ethanol. The sorbed substances were eluted in a step gradient of 10, 30, 40, and 96% aqueous ethanol. To isolate the sum total of water-soluble polyphenols, the 30% aqueous ethanol fraction was concentrated on a rotary evaporator and lyophilized.

Individual polyphenols were isolated by HPLC on an Agilent Technologies 1200 chromatograph with a DAD detector (Waldbronn, Germany), using a semi-preparative column XSelectCSHPrepC18, 5 μL, 10 × 250 mm (Waters, Milford, MA, USA); for rechromatography and analytical purposes, a Phenomenex C18, 5 μL, 4.6 × 250 mm column (Waters, Milford, MA, USA) was used. Solutions: A, 0.1% TFA; B, acetonitrile. Acetonitrile concentration gradient: 0–10 min, 15%; 28 min, 25%; 33–38 min, 60%; 43 min, 15%. Flow rate, 3 mL/min (for analysis and rechromatography, 1 mL/min). Absorption was recorded at 269 nm.

Mass spectrometric analysis of the isolated polyphenols was performed on a Q-TOF LC-MS Agilent Technologies 6520B series instrument (Waldbronn, Germany) under the following conditions: ionization source, ESI; drying gas flow, 5 L/min; drying gas temperature, 300 °C; voltage on skimmer cone and fragmentor, 20 V and 125 V, respectively; mass range, in MS mode and in Targeted MS/MS mode, 100–2000 *m*/*z* and 50–2000 *m*/*z*, respectively; collision energy, 35 and 50 eV; ionization method, negative. Samples were introduced into the mass spectrometer using an Agilent Technologies 1200 series chromatograph, Zorbax SB C18 column, 3 μm, 0.5 × 150 mm. Mobile phase: A, 0.1% formic acid solution; B, acetonitrile + 0.1% formic acid. Elution was performed on an Agilent Technologies 1260 Cap Pump series instrument (Waldbronn, Germany) at a flow rate of 15 μL/min. Concentration gradient of solution B: 0–5 min, 20%; 20 min, 25%; 25 min, 30%; 25.1–30 min, 60%; 35 min, 20%. Solutions were degassed on an Agilent Technologies 1260 μ-degasser (Waldbronn, Germany). Samples were introduced into the column using an Agilent Technologies Micro WPS instrument (Waldbronn, Germany), 1 μL at a time, from a polyphenol solution with a concentration of 0.1 mg/mL.

Identification of compounds based on mass spectrometry data was carried out using the public databases ChemSpider (http://www.chemspider.com) “URL (Accessed: 7 January 2020)”, SciFinderScholar (https://scifinder.cas.org) “URL (Accessed: 8 January 2020)”, KeggLigand (http://www.genome.jp/kegg/) “URL (Accessed: 8 January 2020)”, and Phenol-Explorer (www.phenol-explorer.eu) “URL (Accessed: 9 January 2020)”.

NMR spectroscopy of isolated polyphenols. NMR ^1^ H (600 MHz) and NMR ^13^ C (150 MHz) spectra were recorded on a Varian 600 NMR spectrometer (Varian, Lexington, MA, USA) in DMSO-d_6_ solvent. HMDS was used as an internal standard. MestReNova software v.15.0.0 (Mestrelab Research, SLU, Santiago de Compostela, Spain) was used to record and read the spectra.

Identification of compounds based on NMR spectroscopy data was carried out using the public databases ChemSpider (http://www.chemspider.com) “URL (Accessed: 10 March 2020)”, SDBS (https://sdbs.db.aist.go.jp/) “URL (Accessed: 10 March 2020)”, OdanChem (https://odanchem.org/) “URL (Accessed: 15 March 2020)”, and NMRShiftDB (https://nmrshiftdb.nmr.uni-koeln.de/) “URL (Accessed: 15 March 2020)”.

NMR spectroscopic characteristics of polyphenols of Rutan are presented below.

**R5.** Off-white to yellow-tinted solid. (-) ESI-MS m/z 939.11 [M-H]^−^, MW 940, formula C_41_H_32_O_26_.

**^1^H-NMR (600 MHz, DMSO-*d*_6_)**: *δ* 7.10 (s, H-F 2′, 6′), 7.04 (s, H-B 2′, 6′), 6.97 (s, H-E 2′, 6′), 6.94 (s, H-C 2′, 6′), 6.89 (s, H-D 2′, 6′), 6.26 (d, *J* = 8.3 Hz, H-1), 5.57 (d, *J* = 7.1 Hz, H-2), 5.89 (t, *J* = 7.3 Hz, H-3), 5.61 (t, *J* = 7.1 Hz, H-4), 4.38 (m, overlapped, H-5), 4.50 (d, *J* = 7.5 Hz, H-6), 4.37 (d, *J* = 7.2 Hz, H-6).

**^13^C-NMR (150 MHz, DMSO-*d*_6_)**: ***δ***

Sugar-A: 93.8 (C-1), 74.4 (C-5), 74.1 (C-3), 72.2 (C-2), 69.8 (C-4), 63.1 (C-6);

B: 166.2 (C-7′), 146.5 (C-3′, 5′), 140.7 (C-4′), 119.7 (C-1′), 110.6 (C-2′, 6′);

C: 166.99 (C-7′), 146.3 (C-3′, 5′), 140.3 (C-4′), 120.2 (C-1′), 110.4 (C-2′, 6′);D: 167.3 (C-7′), 146.25 (C-3′, 5′), 140.08 (C-4′), 120.4 (C-1′), 110.38 (C-2′, 6′);

E: 166.9 (C-7′), 146.4 (C-3′, 5′), 140.32 (C-4′), 120.21 (C-1′), 110.5 (C-2′, 6′);

F: 167.9 (C-7′), 146.44 (C-3′, 5′), 139.97 (C-4′), 120.0 (C-1′), 110.34 (C-2′, 6′);

**HMBC (C): *δ***

Sugar-A: 74.4/166.2 (C-1), 93.8/74.1/166.99 (C-2), 74.1/69.8/167.3 (C-3), 166.9/63.1/74.1 (C-4), 63.8/69.8 (C-5), 74.4/69.8/167.9 (C-6);

B: 166.2/119.7/140.7/146.5/110.6 (C-2′, 6′);

C: 120.2/166.99/140.3/146.3/110.4 (C-2′, 6′);D: 167.3/120.4/140.08/146.25/110.38 (C-2′, 6′);

E: 166.9/120.21/140.32/146.4/110.5 (C-2′, 6′);

F: 167.9/120.0/139.97/146.44/110.34 (C-2′, 6′).

**R6**. Off-white to yellow-tinted solid. (-) ESI-MS *m*/*z* 1091.4278 [M-H]^−^, MW 1092, formula C_48_H_36_O_30_.

**^1^H-NMR (600 MHz, DMSO-*d*_6_)**: ***δ*** 7.26 (d, *J* = 2.3 Hz, H-D 5′), 7.20 (s, H-E 2′, 6′), 7.16 (d, *J* = 2.3 Hz, H-D 2′, 6′), 7.11 (s, H-F 2′, 6′), 7.06 (s, H-B 2′, 6′), 7.00 (s, H-C 2′, 6′), 6.97 (s, H-G 2′, 6′), 6.26 (d, *J* = 8.3 Hz, H-1), 5.95 (t, *J* = 9.6 Hz, H-3), 5.65 (m, H-4), 5.62 (m, H-2), 4.55 (m, H-6), 4.43 (m, H-5), 4.37 (m, H-6).

**^13^C-NMR (150 MHz, DMSO-*d*_6_)**: ***δ***

Sugar-A: 93.8 (C-1), 74.38 (C-3), 74.3 (C-5), 72.17 (C-2), 69.8 (C-4), 63.13 (C-6);

B: 166.19 (C-7′), 146.53 (C-3′, 5′), 140.75 (C-4′), 119.7 (C-1′), 110.62 (C-2′, 6′);

C: 166.95 (C-7′), 146.57 (C-3′, 5′), 140.52 (C-4′), 120.14 (C-1′), 110.49 (C-2′, 6′);D: 166.51 (C-7′), 151.59 (C-2′), 147.44 (C-4′), 144.76 (C-3′), 133.0 (C-1′), 115.12 (C-5′), 117.5 (C-6′)

E: 166.63 (C-7′), 146.38 (C-3′, 5′), 140.35 (C-4′), 120.2 (C-1′), 110.84 (C-2′, 6′);

F: 167.93 (C-7′), 146.45 (C-3′, 5′), 139.99 (C-4′), 121.01 (C-1′), 110.44 (C-2′, 6′);

G: 167.03 (C-7′), 146.47 (C-3′, 5′), 140.41 (C-4′), 120.35 (C-1′), 110.33 (C-2′, 6′);

**HMBC (C): *δ***

Sugar-A: 74.4/166.2 (C-1), 93.8/167.02 (C-2), 74.1/69.8/166.51 (C-3), 166.95/74.38 (C-4), 63.13/69.8 (C-5), 74.3/69.8/167.93 (C-6);

B: 166.19/119.70/140.75/146.53/110.62 (C-2′, 6′);

C: 120.2/166.95/140.52/146.57/110.49 (C-2′, 6′); D: 166.51/117.50/139.99/144.76/147.44 (C-5′), 166.51/115.12/139.99/144.76/147.44 (C-6′);

E: 166.63/120.20/140.35/146.38/110.84 (C-2′, 6′);

F: 167.93/121.01/139.99/146.45/110.44 (C-2′, 6′);

G: 167.02/120.35/133.0/140.41/146.47/151.59 (C-2′, 6′).

**R7.** Off-white to yellow-tinted solid. (-) ESI-MS *m*/*z* 1243,1315 [M-H]^−^, MW 1244, formula C_55_H_40_O_34_.

**^1^H-NMR (600 MHz, DMSO-*d*_6_)**: ***δ*** 7.52 (d, *J* = 2.3 Hz, H-H 6′), 7.42 (d, *J* = 2.3 Hz, H-H 5′), 7.24 (d, *J* = 3.2 Hz, H-D 6′), 7.23 (s, H-G 2′, 6′), 7.15 (d, *J* = 3.2 Hz, H-D 5′), 7.09 (s, H-F 2′, 6′), 7.03 (s, H-B 2′, 6′), 6.97 (s, H-C 2′, 6′), 6.94 (s, H-E 2′, 6′), 6.23 (d, *J* = 8.3 Hz, H-1), 5.92 (m, H-3), 5.62 (m, H-4), 5.59 (m, H-2), 4.52 (m, H-6), 4.40 (m, H-5), 4.34 (m, H-6).

**^13^C-NMR (150 MHz, DMSO-*d*_6_)**: ***δ***

Sugar-A: 93,79 (C-1), 74,41 (C-3), 74,37 (C-5), 72,17 (C-2), 69,8 (C-4), 63,13 (C-6);

B: 166.19 (C-7′), 146.44 (C-3′, 5′), 140.74 (C-4′), 110.62 (C-2′, 6′), 119.71 (C-1′);

C: 166.95 (C-7′), 145.13 (C-3′, 5′), 140.33 (C-4′), 120.45 (C-1′), 110.49 (C-2′, 6′);

D: 166.47 (C-7′), 151.02 (C-2′), 145.18 (C-4′), 139.88 (C-3′), 133.16 (C-1′), 117.36 (C-5′), 115.22 (C-6′); E: 167.01 (C-7′), 146.47 (C-3′, 5′), 140.23 (C-4′), 120.15 (C-1′), 110.33 (C-2′, 6′);

F: 167.92 (C-7′), 146.61 (C-3′, 5′), 139.98 (C-4′), 121.01 (C-1′), 110.44 (C-2′, 6′);

G: 166.64 (C-7′), 146.53 (C-3′, 5′), 140.51 (C-4′), 120.41 (C-1′), 110.84 (C-2′, 6′);

H: 165.88 (C-7′), 151.56 (C-2′), 145.13 (C-4′), 139.98 (C-3′), 132.89 (C-1′), 118.21 (C-5′), 115.47 (C-6′);

**HMBC (C): *δ***

Sugar-A: 72.17/166.19 (C-1), 93.79/166.95 (C-2), 72.17/69.80/166.47 (C-3), 167.02/63.13/74.37 (C-4), 69.80 (C-5), 74.37/69.80/167.92 (C-6);

B: 166.19/119.71/146.44/140.74/110.62 (C-2′, 6′);

C: 120.14/166.95/140.33/145.13/110.49 (C-2′, 6′);

D: 115.22/139.98/166.47/144.66 (C-5′), 117.36/145.18/144.66/139.98/166.47 (C-6′); E: 132.89/120.15/151.56/140.23/146.47/167.02/110.33 (C-2′, 6′);

F: 167.91/121.01/139.98/146.61/110.44 (C-2′, 6′);

G: 166.64/120.41/140.51/146.53/133.16/151.02 (C-2′, 6′);

H: 165.88/115.47 (C-5′), 118.21/139.98/145.13 (C-6′).

#### 4.2.2. Geraniin (Ellagitannin of the Aerial Parts of *Geranium sanguineum* L.)

Crushed air-dried aerial parts of *G. sanguineum* (10 g) were placed in an extractor equipped with a steam jacket and a reflux condenser, 200 mL 40% aqueous ethanol was added, and this heated to a temperature of 55–60 °C for 2 h. The extraction process was repeated three times. The extracts were concentrated on a vacuum evaporator (50 °C, 300–350 mm Hg). When the volume of the still residue reached ~313 mL, the thickening was stopped and successive treatment with chloroform and ethyl acetate was carried out. To ensure complete extraction of lyophilic substances, chloroform treatment was carried out thrice (for 15 min every time), and the mixture was then left until complete separation of the phases. The chloroform layer was poured into a collector. The resulting aqueous residue was treated three times with ethyl acetate for complete extraction of phenolic compounds. The ethyl acetate extract was dried with anhydrous Na_2_SO_4_ for 8 h. After decantation, thickening was carried out on a vacuum evaporator at 50 °C and 350 mm Hg. When the volume of the still residue reached ~25 mL, the thickening was stopped and the polyphenols were precipitated with chloroform from the ethyl acetate concentrate. The resulting suspension was left for 2 h until sedimentation was complete. Filtration was carried out on a Nutsche filter through a layer of coarse calico and a layer of filter paper. The suspension of polyphenols was filtered under vacuum, the sediment was washed with 20 mL chloroform. The sediment from the filter was transferred to trays with parchment paper and dried under a hood for 4–5 h until the chloroform smell completely disappeared.

To obtain geraniin, hydrophobic chromatography of the precipitate was performed on a column (50 × 10 mm) with Silochrom 80 C18 sorbent. The column was equilibrated with 0.1% TFA at a flow rate of 2 mL/min. The extract containing polyphenols (200 mg) was dissolved in 20 mL 0.1% TFA and applied onto the column. The column was washed with 50 mL of the same solution. The sorbed substances were eluted with 300 mL of 7.5% ethanol solution in 0.1% TFA (the column was then regenerated with 96% ethanol). The resulting eluate was concentrated on a rotary evaporator and lyophilized. Geraniin was isolated by semi-preparative HPLC on an Agilent Technologies 1200 chromatograph with a DAD detector. A Zorbax 300SB C18 5 μm 7.6 × 250 mm column was used. Mobile phase: solution A, 0.1% TFA; solution B, acetonitrile. Acetonitrile concentration gradient (%/min): 10%/1 min, 15%/40 min, 60%/45 min, 12%/46 min. Flow rate, 2.5 mL/min. Absorption was recorded at 269 nm (reference 360 nm).

Mass spectrometric analysis of geraniin and its identification by mass spectrometry data were carried out as described in Section 4.2.1.

Crystallization of geraniin. Single crystals of geraniin were obtained by dissolving 20 mg of the compound in 1 mL of an ethanol–water mixture (1:1, *v*/*v*). The solution was gently heated to 50 °C to ensure complete dissolution and then left to slowly evaporate at room temperature under static conditions. After 48 h, fine needle-like pale yellow crystals were formed. From the resulting batch, single crystals suitable for single-crystal X-ray diffraction (SCXRD) analysis were carefully selected.

X-ray diffraction analysis. Single-crystal X-ray diffraction data for geraniin were collected at 290 K using an XtaLAB Synergy diffractometer equipped with a micro-focus sealed X-ray tube (PhotonJet, Cu Kα radiation, λ = 1.54184 Å) and a HyPix-3000 detector. A mirror monochromator was employed, and data were acquired using ω-scan mode with a detector resolution of 10.0000 pixels/mm. Data reduction was performed using the *CrysAlis^Pro^* 1.171.41.123a software package (Rigaku Oxford Diffraction, UK). The structure was solved by direct methods using the *SHELXT* (2018/2) software package and refined by full-matrix least squares on F^2^ using *SHELXL* (2016/6) [154]. All non-hydrogen atoms were refined using anisotropic displacement parameters. Hydrogen atoms were positioned geometrically and refined using a riding model. Molecular graphics were generated using the OLEX2 software package [155] (OlexSys, UK).

Hirschfeld Surface Analysis. The Hirschfeld surface and corresponding fingerprint plots for the title compound were analyzed using *Crystal Explorer* [122], based on the crystal structure information file (CIF) [156]. Two-dimensional fingerprint plots were generated using the standard range 0.6–2.6 Å, with the distances *d***_e_** (external) and *d***_i_** (internal) shown on the plot axes. The *d*_norm_ function represents a normalized distance, calculated as the ratio of the distances from a surface point to the nearest interior (*d*_i_) and exterior (*d*_e_) atoms, relative to the van der Waals (vdW) radii of the respective atoms. The Hirshfeld surfaces were visualized as three-dimensional *d*_norm_ maps using high-resolution surface settings. The surfaces were color-coded using a scale from red (high interaction) to blue (low interaction), corresponding to *d*_norm_ values ranging from −0.728 (red) to 1.428 (blue). Regions in red highlight close contacts (significant intermolecular interactions), green indicates contacts around the van der Waals separation, and blue corresponds to regions with negligible intermolecular interactions.

### 4.3. Evaluation of In Vitro Antiviral Activity in Permissive Cell Cultures

#### 4.3.1. General Principles

Viruses and cells were obtained, respectively, from the State Collection of Viruses and the Russian Collection of Cell Cultures of the Ivanovsky Institute of Virology (a division of the Gamaleya Research Center for Epidemiology and Microbiology of the Russian Ministry of Health).

In all cases, DMSO was used to prepare stock solutions of the polyphenols under study and reference compounds. Working solutions were prepared by diluting stock solutions with appropriate culture media; the final DMSO concentration did not exceed 1%. Reference compounds served as a positive control; working solutions without polyphenols and reference compounds were used as a negative control.

In each virus–cell system, cytotoxicity ranges were determined for polyphenols and reference compounds using MTT assay. Half-maximal cytotoxic concentrations (CC_50_) were determined from the curves of the dependence of the proportion of cells retaining viability after incubation with the compound (relative to the number of cells incubated in the absence of the compound) on its concentration, using nonlinear regression equations (4 parameters).

Antiviral activity was studied at concentrations outside the established cytotoxicity ranges. The activity was assessed by the difference between the levels of virus reproduction in the absence (control) and presence of the drug (experiment)

A = A_c_ − A_e_,

where A_c_ and A_e_ are the levels of virus reproduction in the control and experiment samples, respectively. For each concentration, the coefficient of inhibition of virus reproduction (CI), or protection index, was calculated:

CI = [(A_c_ − A_e_)/A_c_] × 100 (%) (1)

The half-maximum inhibitory concentration of the compounds (IC_50_) was determined from the curves of CI dependence on compound concentration, using nonlinear regression equations (4 parameters). The selectivity index (SI) was calculated using the following formula:

SI = CC_50_/IC_50_ (2)

#### 4.3.2. IAV and SARS-CoV-2

Stocks of both viruses were prepared as described earlier [157]. The effect of polyphenols on the reproduction of IAV and SARS-CoV-2 viruses was studied as described previously [158]. Oseltamivir (Sigma-Aldrich, USA) and nilmatrevir (Zenji Pharmaceuticals, Huai’an, China) were used as reference compounds. Since the effect on reproduction was judged by the difference in virus titers, expressed in logarithms Δ(lg TCID_50_), the maximum values of this parameter for each polyphenol and reference compound were included in Table 3 and Table 4.

#### 4.3.3. HIV-1

HIV-1 stock was prepared according to the standard protocol [159]. The effect of polyphenols on the reproduction of HIV-1 was studied as described previously [160]. Azidothymidine (Sigma-Aldrich, USA) was used as a reference compound.

#### 4.3.4. HSV-1 and HSV-2

HSV-1 and HSV-2 stocks were prepared in Vero cells; the virions were concentrated by centrifugation of the conditioned medium (14,000 *g*). The titer of viral stocks was determined by the method of Sarisky et al. [161]. The effect of polyphenols on the reproduction of HSV-1 and HSV-2 was studied as described previously [162,163]. Foscarnet (Sigma-Aldrich, USA) was used as a reference compound. In addition to IC_50_, maximal inhibitory concentrations (IC_95_) were determined for each polyphenol and reference compound. The index 95 (and not 100) was used for two reasons: (1) CPEs were observed in 95–100% of the infected cells not exposed to polyphenols or foscarnet; and (2) in cultures of uninfected cells not exposed to polyphenols or foscarnet, ~5% lost viability by the end of the incubation period (after 48 h).

#### 4.3.5. CMV

##### Cells

Diploid HELF was cultured in DMEM medium supplemented with 10% fetal calf serum (Biolot, Russia), 2 mM L-glutamine, and 50 μg/mL gentamicin (both purchased from Gibco, USA). The cells were incubated in a CO_2_ incubator (Sanyo, Tokyo, Japan) at 37 °C in an atmosphere of 5% CO_2_. The cell culture was not contaminated with mycoplasmas, as determined using MycoReport PCR kits (Eurogen, Russia).

##### Virus

The reference CMV strain AD169 was propagated in the culture of human embryonic lung fibroblasts (HELF). Virions were collected by centrifugation (14,000 *g*). The infectious titer of CMV was determined by a modified plaque assay [164] and expressed as the amount of PFU contained in 1 mL [129]. A virus with an infectious titer of at least 5 × 10^5^ PFU/mL was used in this work.

##### Cytotoxicity Determination

Cytotoxicity was measured by the standard MTT method as described [125]. Briefly, HELF cells were cultivated in 48-well culture plates with serial dilutions of each compound for 72 h at 37 °C in 5% CO_2_ atmosphere. Thereafter, the medium was replaced with 200 µL of a 1 mg/mL solution of MTT [3-(4,5-dimethylthiazol-2-yl)-2,5-diphenyl tetrazolium bromide) in DMEM. The cells were incubated at 37 °C for 3 h, and 200 µL of 0.1 M HCl solution in 2-propanol was added to each well. After a few minutes of incubation at room temperature (to ensure that all crystals were dissolved), the plates were read using an automatic plate reader (TECAN, Mannedorf, Switzerland) with a 570 nm test wavelength and a 690 nm reference wavelength. The 50% cytotoxic concentration (CC_50_) was defined as the compound concentration that caused a 50% reduction in the number of viable cells.

##### Immunocytochemical Detection of Infected Cells

To detect infected cells and virus-induced plaques, experimental and control cultures were stained with a mixture of monoclonal antibodies (MAbs) to the immediate early IE1-72 (Santa Cruz Biotechnology, Santa Cruz, CA, USA) and early pp65 (Abcam, Cambridge, UK) CMV proteins. On day 5 after the infection, the cells were fixed with chilled methanol for 20 min. Then the mAb mixture was applied, followed by incubation at 37 °C for 1 h. The result of the immunocytochemical reaction was developed using anti-mouse antibodies conjugated with horseradish peroxidase (ImmunoResearch, West Grove, PA, USA). A 0.5 mg/mL solution of diaminobenzidine (AppliChem, Darmstadt, Germany) with 0.03% hydrogen peroxide (Reakhim, Staraya Kupavna, Russia) was used as a substrate. The reaction was stopped by washing the cell monolayer with distilled water. The stained preparations were examined under a light microscope (Olympus, Tokyo, Japan) at 400× to count 98i, the infected cells, and virus-induced plaques.

##### Determination of Antiviral Activity

Two experimental designs were used.

*Pre-inoculation setup.* DMEM maintenance medium containing the polyphenols (500 μL) was applied to the HELF monolayers, and the cells were incubated for 1 h at 37 °C in an atmosphere of 5% CO_2_. Then the CL was collected, the cells were washed with pure DMEM medium 3 times, the virus was added to a final titer of 300 PFU/mL, and the cells were incubated for 1 h at 37 °C in an atmosphere of 5% CO_2_. After the incubation, the cell monolayer was washed with pure DMEM medium 3 times, DMEM maintenance medium was added, and the incubation was resumed.

*Post-inoculation setup*. The monolayer of HELF was infected with the virus to a final titer of 300 PFU/mL. After virus adsorption for 1 h at 37 °C in an atmosphere of 5% CO_2,_ the monolayer was washed with pure DMEM medium 3 times, DMEM maintenance medium containing the polyphenols (500 μL) was added immediately thereafter, and the incubation was resumed.

After 5 days of incubation, the number of cells containing viral proteins (stained with virus-specific monoclonal antibodies) and the number of virus-induced plaques were determined as described in Section 4.3.5.

To assess the effect on the production of infectious virions, CM was collected from the cells exposed to polyphenols (in the post-inoculation setup) and introduced into uninfected HELF cultures, which were then incubated for 5 days. The infectious activity of the virus in experimental and control samples was judged by the number of virus-specific plaques formed.

The CI and IC_50_ values were determined using formulas (1) and (2) given in Section 4.3.1. IC_50_ values were calculated from the curves of CI dependence on the concentration. Ganciclovir (Roche, Switzerland), a drug recommended for the treatment of CMV infection, was a reference compound.

#### 4.3.6. EBV

The virus–cell system used was the B-lymphoblastoid EBV-positive cell line B95-8, one of distinctive characteristics of which is the ability to maintain EBV replication without loss of viability; this phenomenon is called aberrant latent infection [165,166]. The cells were cultured in a mixture of RPMI-1640, DMEM and Eagle’s MEM, taken in equal volumes, and 10% fetal calf serum (Biolot, Russia).

To assess cytotoxicity, B95-8 cells (5 × 10^5^ per 1 mL) were incubated with polyphenols at 37 °C in an atmosphere of 5% CO_2_ for 72 h. The experiment was then carried out as described in Section 4.3.5.

To assess the activity against EBV, B95-8 cells (5 × 10^5^ per 1 mL) were incubated with polyphenols at 37 °C in an atmosphere of 5% CO_2_ for 48 h. Then, the cells were pelleted by centrifugation (14,000 *g*), and EBV DNA levels in supernatants were determined by quantitative real-time polymerase chain reaction (RT-PCR). Ganciclovir from Roche (Switzerland), which has antiviral activity against EBV, was used as a reference compound. The concentration at which the amount of EBV genomic DNA decreased by 50% relative to the control was measured as IC_50_.

DNA extraction was performed using the DNA-sorb-V kit (Interlabservice, Russia), according to the manufacturer’s instructions. RT-PCR was performed using a reagent kit for the detection and quantitative determination of EBV, CMV, and herpes virus type 6 (HHV6-A/B) DNA in clinical material (AmpliSens^®^ EBV/CMV/HHV6-screen-FL Detection and quantitative determination of EBV, CMV, and HHV6 DNA) from the same manufacturer. The *GAPDH* gene was used as an endogenous internal control. The detection limit corresponded to 1 copy per 1 reaction.

### 4.4. Statistical Analysis

All experiments were performed in triplicate at least three times independently. The values are presented as the means ± standard deviations (S.D.). The statistical significance of differences between the two groups was assessed by Student’s *t*-test using GraphPad Prism 8 (GraphPad Software Inc., Boston, MA, USA). Multiple comparisons were evaluated by analysis of variance (ANOVA) with Dunnett’s post hoc test. A *p*-value of ≤0.05 was considered significant.

## 5. Conclusions

In this study, we have demonstrated for the first time that sumac polyphenols are capable of suppressing—in addition to HIV, influenza virus, and SARS-CoV-2—the reproduction of representatives of the Alphaherpesvirinae, Betaherpesvirinae, and Gammaherpesvirinae subfamilies of the family Orthoherpesviridae. This finding may have important fundamental implications. It remains for further studies to determine whether any sumac polyphenol qualifies as a pan-herpesvirus inhibitor and what mechanisms underlie the effects observed.

We have also confirmed the activity of geraniin against several unrelated viruses (influenza A virus, HIV-1, SARS-CoV-2, HSV-1, and HSV-2) and reported for the first time that the sumac polyphenols 2,4-bis-O-galloyl-1,3,6-tri-O-galloyl-β-D-glucose (R7) and 2,3,4-bis-O-galloyl-1,6-di-O-galloyl-β-D-glucose (R8) suppress in vitro reproduction of influenza A virus, HSV-1, and HSV-2.

Thus, all active compounds identified meet the criteria for further development as broad-spectrum antivirals. As regards Rutan, an approved drug against influenza and COVID-19 in Uzbekistan, our current results are indicative of its significant promise for the treatment of immunocompromised patients infected with HSV, CMV, or EBV, which resist standard treatment regimens.

## Figures and Tables

**Table 1:** Polyphenols investigated.

Substance	Description	Spectrum of Antiviral Activity	Approved for Diseases (Country)
Rutan *	The sum of water-soluble polyphenols of sumac (*Rhus coriaria*)	Influenza viruses [43], SARS-CoV-2 [44,45,46]	Influenza (Uzbekistan), COVID-19 (Uzbekistan) [46]
R5 (Rutan polyphenol)	1,2,3,4,6-penta-O-galloyl-β-D-glucose	Influenza viruses [67,68,69,70,71,72], SARS-CoV-2 [46,73,74,75,76,77,78,79,80], rhinoviruses [81], respiratory syncytial virus [82], HIV [83,84], rabies virus [85], Zika virus [28,86,87], hepatitis B virus (HBV) [88,89], HCV [28,86,90,91], HSV-1 [92,93], HSV-2 [84], varicella-zoster virus (VZV) [94,95]	-
R6 (Rutan polyphenol)	3-bis-O-galloyl-1,2,4,6-tetra-O-galloyl-β-D-glucose	SARS-CoV-2 [46]	-
R7 (Rutan polyphenol)	2,4-bis-O-galloyl-1,3,6-tri-O-galloyl-β-D-glucose	SARS-CoV-2 [46]	-
R8 (Rutan polyphenol)	2,3,4-bis-O-galloyl-1,6-di-O-galloyl-β-D-glucose	SARS-CoV-2 [46]	-
Geraniin	Polyphenol of *Geranium sanguineum*	Influenza viruses [69,72,96], SARS-CoV-2 [80,97,98,99,100,101], rhinovirus [102], respiratory syncytial virus [102], metapneumovirus [102], HIV [103,104], dengue virus [105,106,107], Zika virus [107], HBV [108,109,110], HCV [111], HSV-1 [112,113,114], HSV-2 [112,115,116,117], enterovirus 71 [118]	Diarrhea (Japan) [119]

* Not to be confused with the product of Aquia Quimica Innovativa (a Brazilian cosmetics manufacturer), which markets rutinyl succinate under the name Rutan SP [https://aqia.net/en-us/produtos/rutan-sp/; site access date 7 August 2025]. The full list of Rutan constituents is given in Table S1.

**Table 2:** Chromato-mass-spectrometric characteristics of Rutan polyphenols.

Substance	Rt	[M-H]^−^	Fragmentation								
	min	*m*/*z*. (%)	*m*/*z*. (%)								
R5	14.54	939.05 (5)	769.09 (100)	617.08 (35)	447.04 (16)	313.02 (5)	169.01 (12)	125.0 (5)			
R6	18.33	1091.2 (3)	939.17 (45)	769.12 (100)	617.09 (33)	447.03 (27)	276.9 (16)	169.01 (35)	124.9 (12)		
R7	1 8.97	1243.4 (10)	1091.8 (5)	939.4 (100)	769.13 (45)	617. 08 (20)	447.05 (15)	276.9 (8)	169.8 (38)	124.9 (10)	
R8	20.39	1395.6 (5)	1243.4 (8)	1091.8 (27)	939.3 (33)	769.12 (100)	601.03 (16)	447.03 (28)	276.9 (7)	169.8 (40)	124.9 (10)

**Table 3:** Effect on the reproduction of influenza A virus/California/07/2009 (H1N1) pdm in MDCK cells.

Substance	Δ(lgTCID_50_)_max_	IC_50_, μg/mL	CC_50_, μg/mL	SI
Rutan	2.5	12.44	144.3	11.6
R5	1.5	8.85	86.34	9.8
R6	1.0	>50	93.13	<2
R7	2.75	2.13	191.01	89.7
R8	4.25	0.8	233	291.3
Geraniin	4.5	10.83	288	26
Oseltamivir	6.0	0.02	>10	>500

**Table 4:** Effect of geraniin on the reproduction of SARS-CoV-2 (HCoV-19/Russia/Moscow-PMVL-12/2020, EPI_ SL_572398) in Vero E6 cells.

Substance	Δ(lgTCID_50_)_max_	IC_50_, μg/mL	CC_50_, μg/mL	SI
Geraniin	4.75	5.2	65.4	12.6
Nirmatrelvir	6.0	0.1	>20	>200

**Table 5:** Effect of geraniin on the reproduction of HIV-1 (HIV_IIIB_) in MT-4 cells.

Substance	IC_50_, μg/mL	CC_50_, μg/mL	SI
Geraniin	1.6	41	25.6
Azidothymidine	0.002	3.6	1800

**Table 6:** Antiviral activity of polyphenols in Vero E6 cells infected with reference strains of HSV-1 or HSV-2 (multiplicity of infection, 0.1 PFU/cell).

Substance	CC_50_, μg/mL	HSV-1_L2_			HSV-2_BH_		
		IC_50_, μg/mL	IC_95_, μg/mL	SI	IC_50_, μg/mL	IC_95_, μg/mL	SI
Rutan	172.16	6.25	12.5	28	12.5	25.0	14
R5	187.71	12.5	25.0	15	12.5	25.0	15
R6	69.62	25.0	>25.0	3	31.25	50.0	2
R7	165.00	5.95	8.32	28	8.32	31.25	20
R8	106.00	10.0	31.25	11	31.25	62.5	3
Geraniin	65.92	1.20	1.56	55	0.39	6.25	169
Foscarnet	>125	15.6	62.5	>8	7.80	31.25	>16

**Table 7:** Anti-CMV activity of polyphenols.

Substance	CC_50_, μg/mL	Post-Inoculation Setup				Pre-Inoculation Setup	
		Inhibition of Intracellular Replication		Inhibition of Infectious Virus Production			
		IC_50_, μg/mL	SI	IC_50_, μg/mL	SI	IC_50_, μg/mL	SI
Rutan	170	5.9	28.8	4.0	42.5	3.4	50
R5	150	7.4	20.5	6.4	23.4	2.2	68
Ganciclovir	185	0.22	840	<0.1	>1850	No activity	

**Table 8:** Inhibition by polyphenols of EBV_B95-8_ replication.

Substance	CC_50_, μg/mL	Inhibition on EBV Replication	
		IC_50_, μg/mL	SI
Rutan	63	>1	<63
R5	85	>10	<8.5
R6	>20	-	-
R7	33	-	-
R8	>20	-	-
Geraniin	63	-	-
Ganciclovir	>1000	100	>10

## Data Availability

The raw data supporting the conclusions of this article will be made available by the authors on request.

## Supplementary Materials

The following supporting information can be downloaded at https://www.mdpi.com/article/10.3390/ijms262110398/s1.
