# Supplementary material for: Sumac Polyphenols as Pan-Herpesvirus Inhibitors
Source: Int J Mol Sci. 2025 Oct 26;26(21):10398. doi: 10.3390/ijms262110398 (PMC12607321; doi:10.3390/ijms262110398)
Supplement: Supplementary file 1 [file ijms-26-10398-s001.zip › ijms-3834155-supplementary.pdf]

Table S1. Polyphenols of the pharmacopoeial substance of Rutan

	Molecular weight	Structure	Content, % (*w/w*)
R5	940	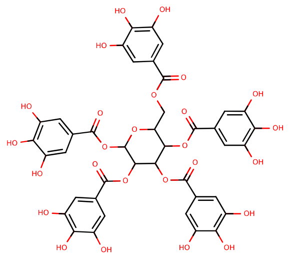	39.2
R6	1092	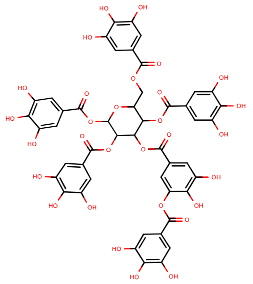	14.8
R7	1244	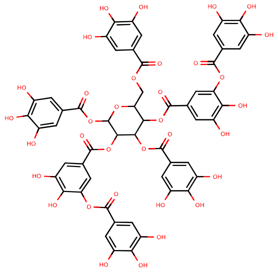	26.3
R8	1396	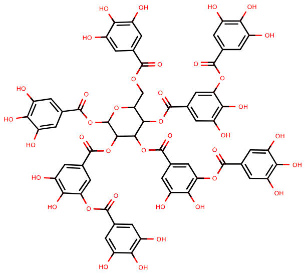	8.4
R9	1548	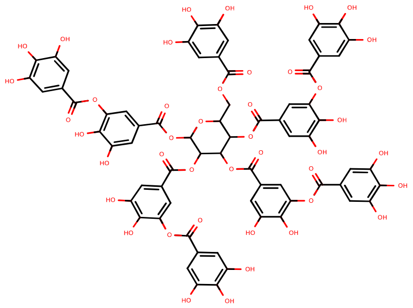	6.7

Mass spectra of polyphenols R5–R8

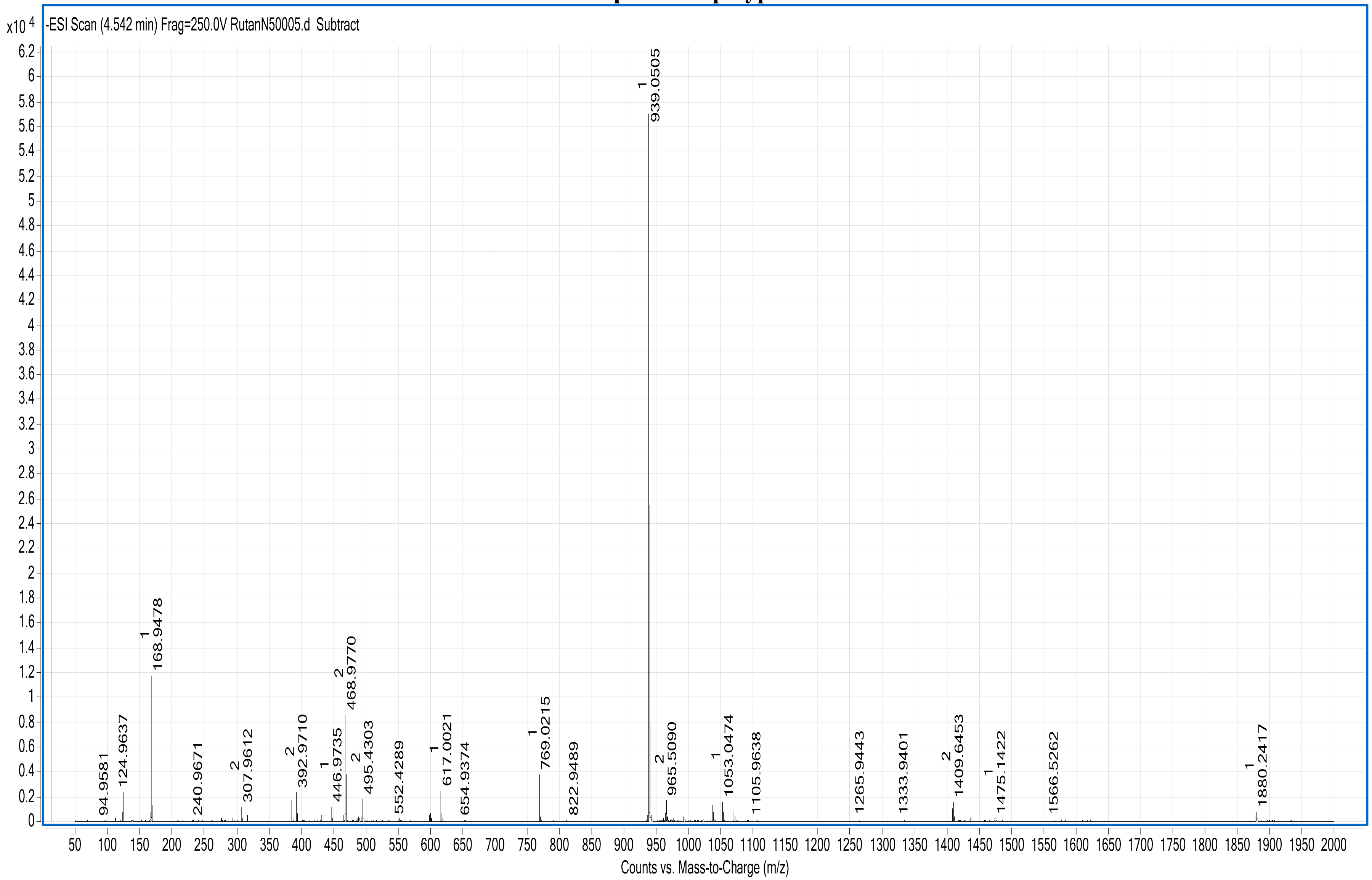

Figure S1. MS spectrum of R5

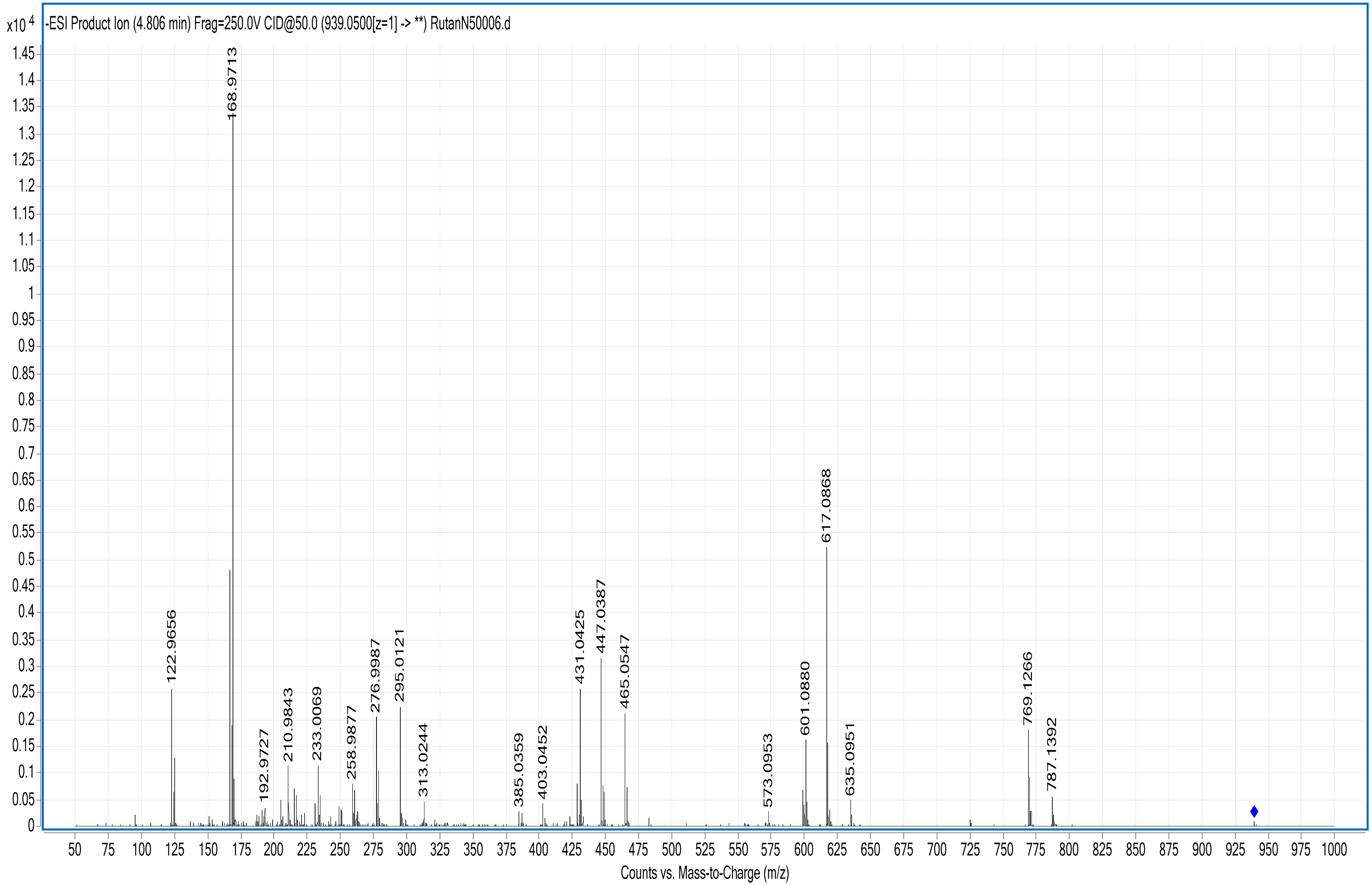

Figure S2. MS/MS spectrum of R5

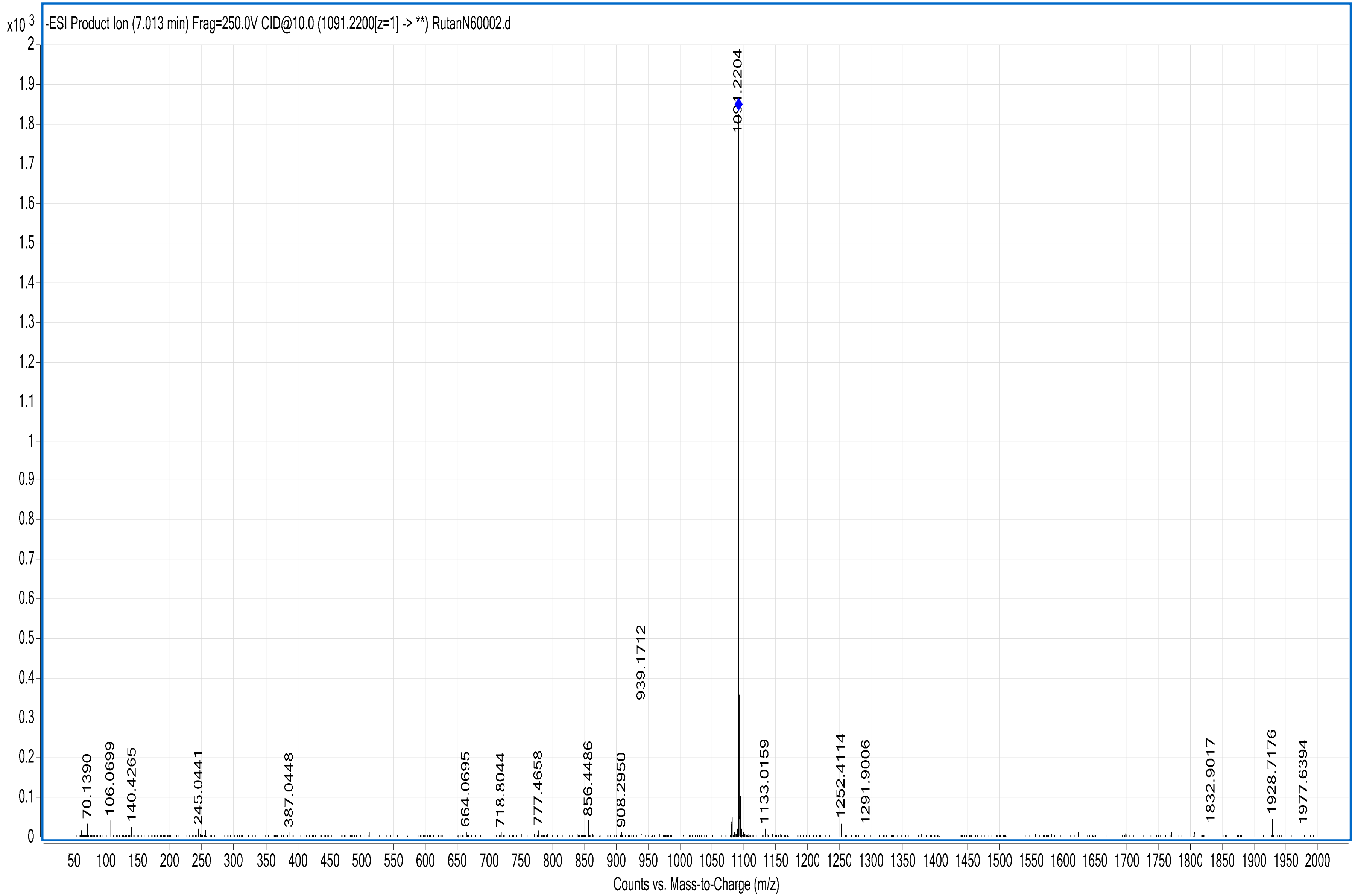

Figure S3. MS spectrum of R6

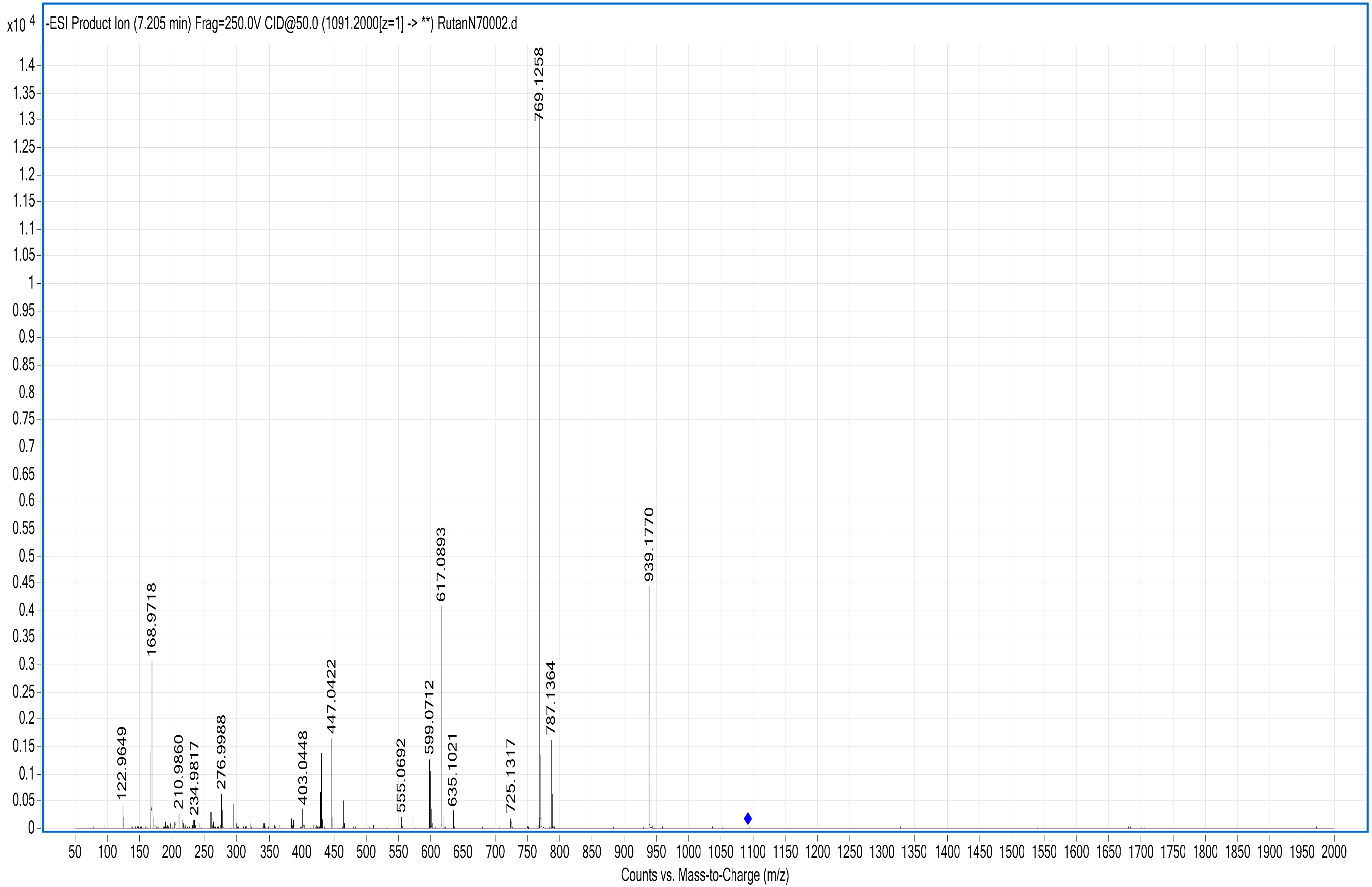

Figure S4. MS/MS spectrum of R6

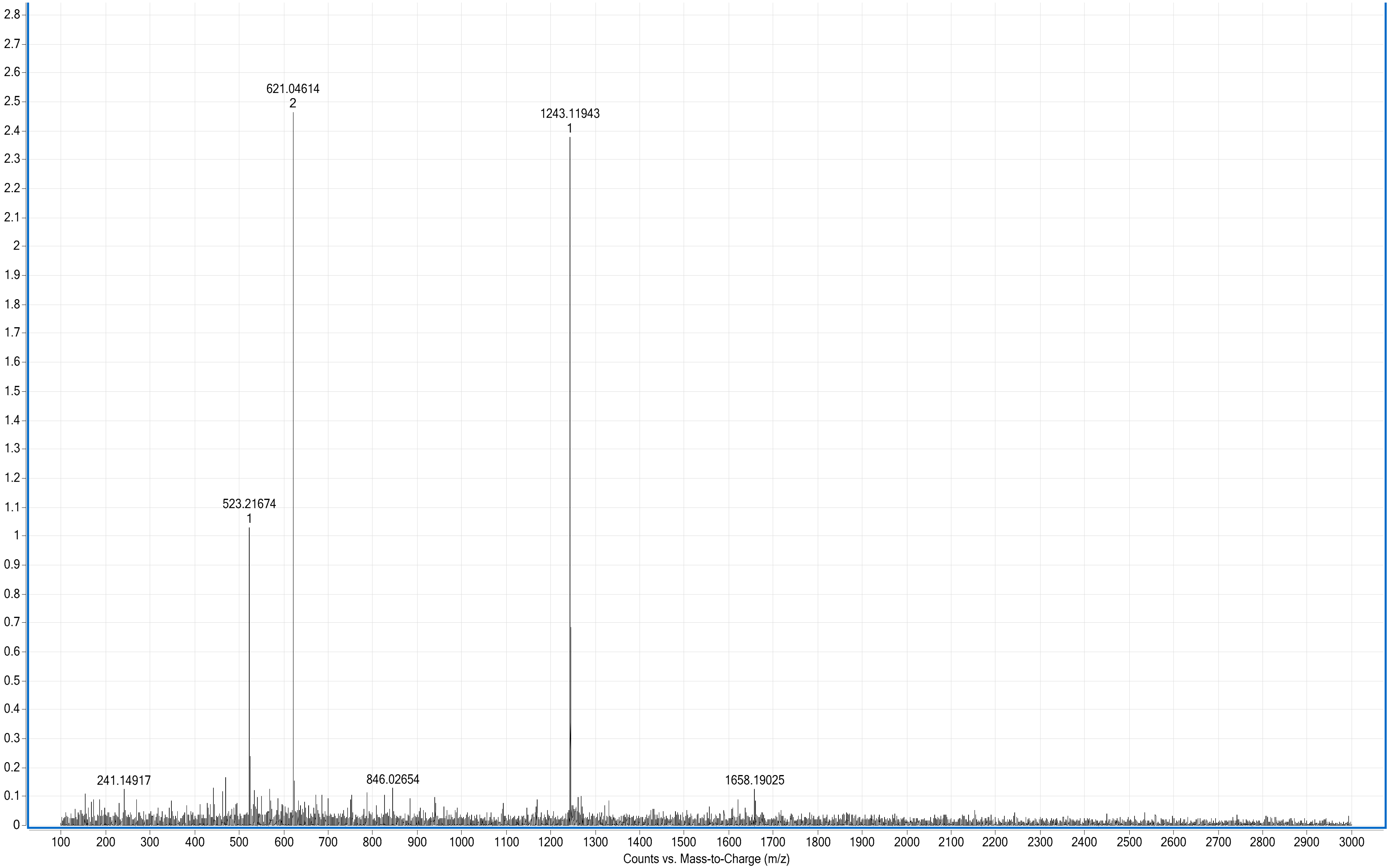

Figure S5. MS spectrum of R7

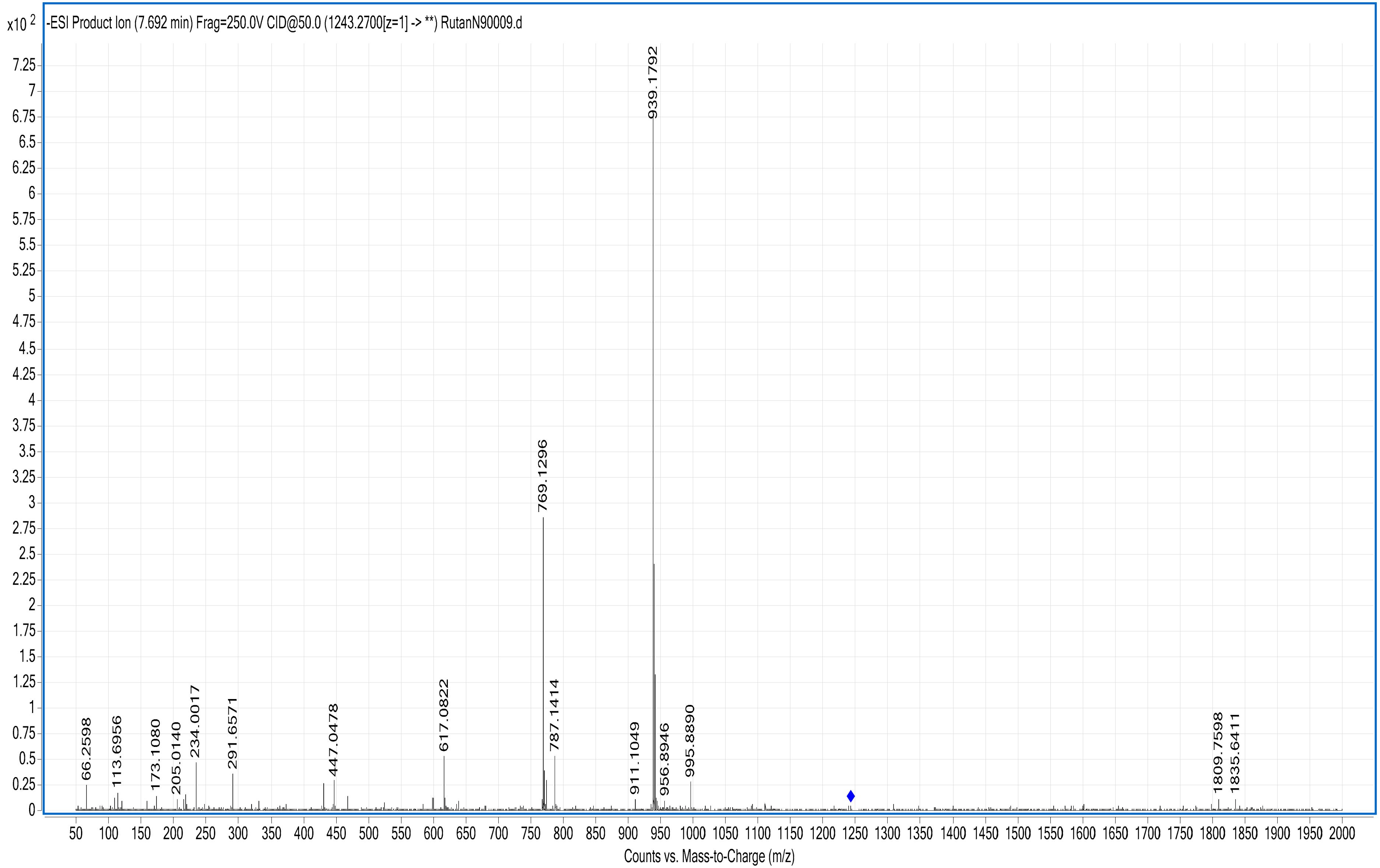

Figure S6. MS/MS spectrum of R7

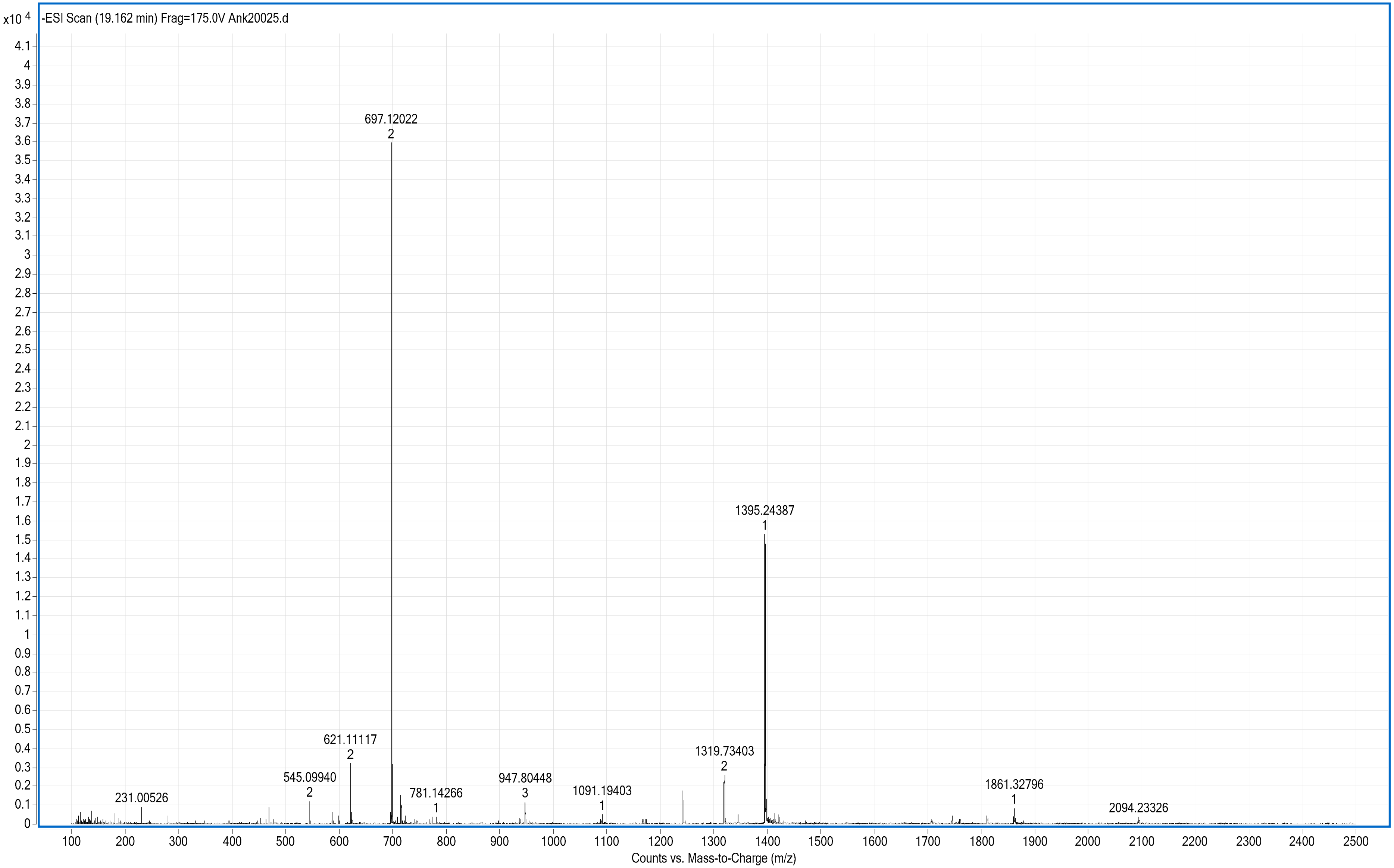

Figure S7. MS spectrum of R8

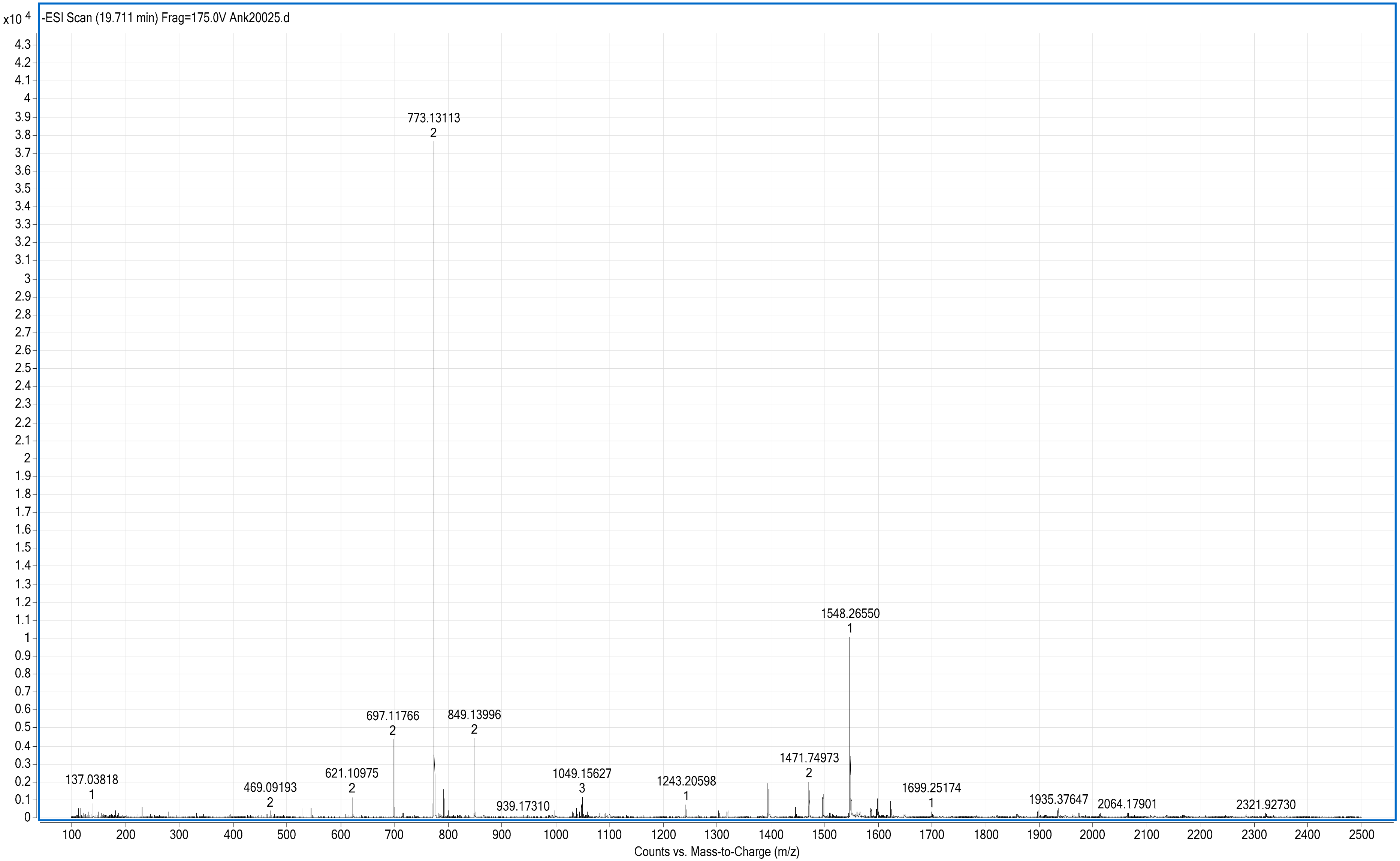

Figure S8. MS spectrum of R9

Mass spectra of geraniin

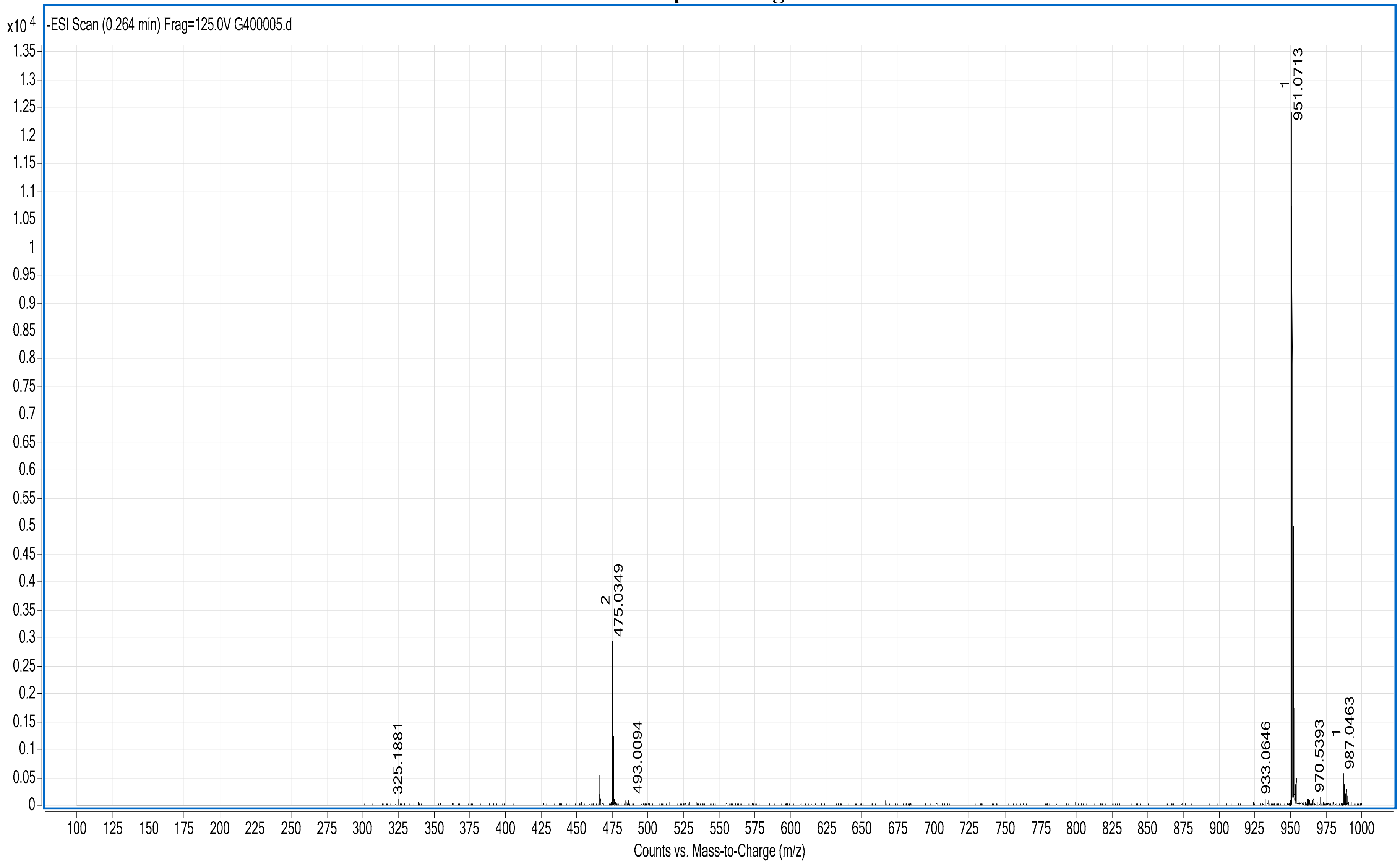

Figure S9. MS spectrum of the molecular ion of geraniin

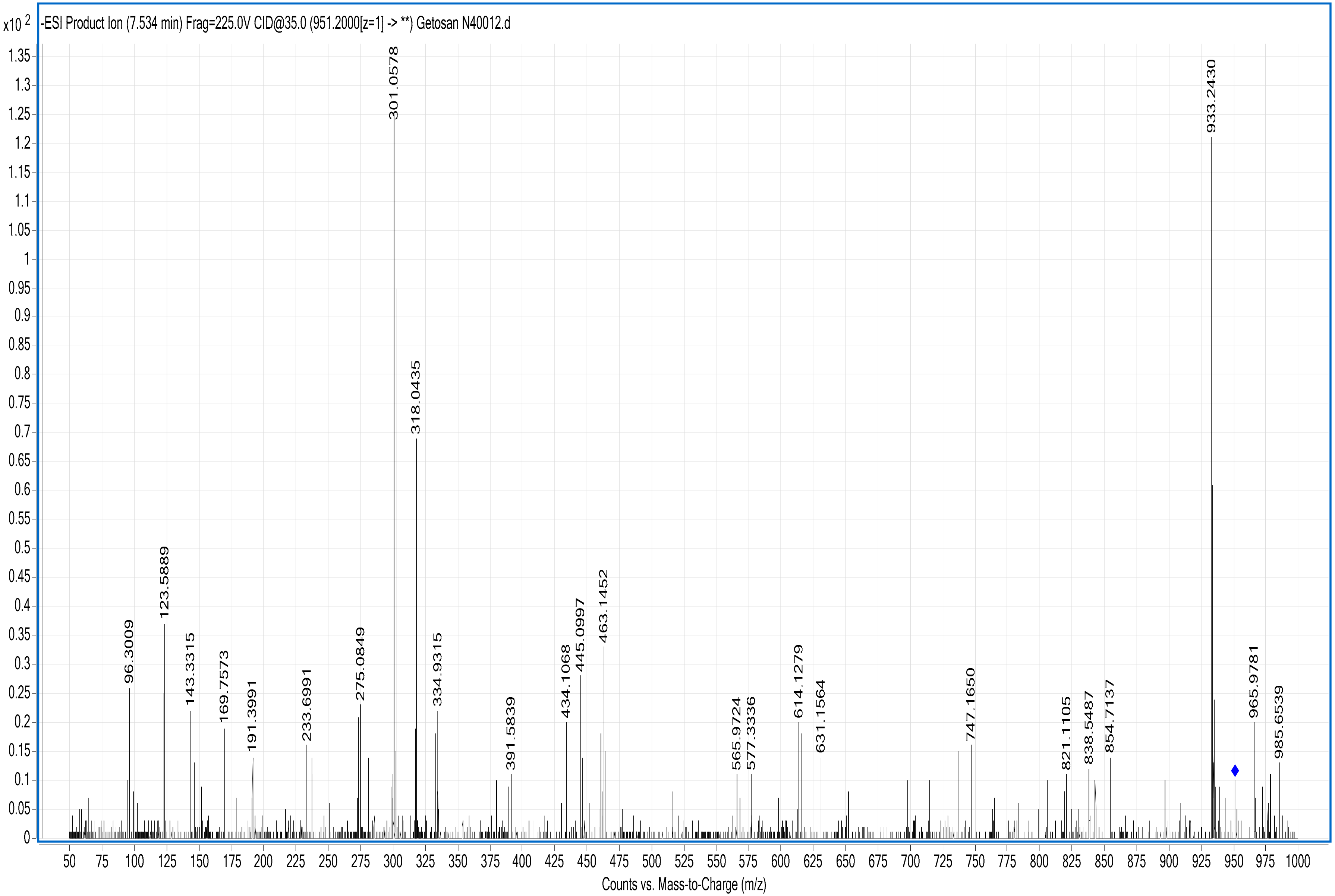

Figure S10. MS and MS/MS spectra of the molecular ion of geraniin
